# Insights into the estimation of surface tensions of mixtures based on designable green materials using an ensemble learning scheme

**DOI:** 10.1038/s41598-023-41448-z

**Published:** 2023-08-29

**Authors:** Reza Soleimani, Amir Hossein Saeedi Dehaghani

**Affiliations:** 1 Department of Chemical Engineering, Faculty of Chemical Engineering, Tarbiat Modares University, P.O. Box 14115-143, Tehran, Iran; 2 Department of Petroleum Engineering, Faculty of Chemical Engineering, Tarbiat Modares University, P.O. Box 14115-143, Tehran, Iran

**Keywords:** Chemical physics, Thermodynamics, Ionic liquids, Theoretical chemistry, Computational chemistry

## Abstract

Precise estimation of the physical properties of both ionic liquids (ILs) and their mixtures is crucial for engineers to successfully design new industrial processes. Among these properties, surface tension is especially important. It’s not only necessary to have knowledge of the properties of pure ILs, but also of their mixtures to ensure optimal utilization in a variety of applications. In this regard, this study aimed to evaluate the effectiveness of Stochastic Gradient Boosting (SGB) tree in modeling surface tensions of binary mixtures of various ionic liquids (ILs) using a comprehensive dataset. The dataset comprised 4010 experimental data points from 48 different ILs and 20 non-IL components, covering a surface tension range of 0.0157–0.0727 N m^−1^ across a temperature range of 278.15–348.15 K. The study found that the estimated values were in good agreement with the reported experimental data, as evidenced by a high correlation coefficient (R) and a low Mean Relative Absolute Error of greater than 0.999 and less than 0.004, respectively. In addition, the results of the used SGB model were compared to the results of SVM, GA-SVM, GA-LSSVM, CSA-LSSVM, GMDH-PNN, three based ANNs, PSO-ANN, GA-ANN, ICA-ANN, TLBO-ANN, ANFIS, ANFIS-ACO, ANFIS-DE, ANFIS-GA, ANFIS-PSO, and MGGP models. In terms of the accuracy, the SGB model is better and provides significantly lower deviations compared to the other techniques. Also, an evaluation was conducted to determine the importance of each variable in predicting surface tension, which revealed that the most influential factor was the mole fraction of IL. In the end, William’s plot was utilized to investigate the model's applicability range. As the majority of data points, i.e. 98.5% of the whole dataset, were well within the safety margin, it was concluded that the proposed model had a high applicability domain and its predictions were valid and reliable.

## Introduction

In the past few years, there has been a surge of interest in ionic liquids (ILs) among scientists, engineers, regulators, and policy makers worldwide^1^. These molten salts, which consist of organic cations and organic/inorganic anions, have gained popularity in various industries as a new class of compounds for diverse applications. Due to their bulky and asymmetrical cation structure^2^, ILs have a low tendency to form an ordered crystal and thus remain in a liquid state at ambient temperature.

The exceptional properties of ILs, such as their good catalytic properties, low vapor pressure, nonflammability, high solvation capacity for various organic compounds, and high thermal and chemical stability, make them promising sustainable alternatives to traditional materials in a wide range of processes^3–5^. ILs are often referred to as “designable materials” because their properties can be tailored for specific processes by making structural modifications to the cation or anion^6^. At present, ILs are being used for various applications, including but not limited to Enhanced Oil Recovery (EOR)^7^ process, extraction processes^8–11^, catalytic reactions^12^, separation processes^13–15^, electrochemistry^16^, lithium batteries^17^, biomass conversion^18^, desulphurization^19^, coal dissolution^20^, bitumen processing^21,22^, crude oil dissolution^23,24^, asphaltene dissolution^25^, and crude oil/water IFT reduction^26^.

Having a comprehensive understanding of the chemical, physical, and thermodynamic properties of ILs or their mixtures with other compounds is crucial, especially since a significant percentage of industrial applications of ILs involve mixtures^27^, such as in EOR processes in reservoirs. This is of great importance from both academic and industrial perspectives.

Surface tension is a critical macroscopic physical property^28^ of ILs and their relevant mixtures. It plays an essential role in the appropriate design and operation of upcoming industrial processes that involve mass transfer, such as distillation, extraction, and absorption^3,29^. In the petroleum industry, surface tension is particularly important for designing fractionators, absorbers, separators, two-phase pipelines, and assessing reservoirs^30^. This is because it significantly affects mass and heat transfer at the interfaces^31^. Interested readers are referred to Tariq et al.^32^ who provide a detailed explanation of why surface tension of ILs is crucial.

Due to the infinite number of possible systems, it is impractical to experimentally measure the surface tension of every possible IL and its mixture with other compounds. Additionally, empirical measurements can be expensive, time-consuming, and susceptible to non-negligible uncertainties^33^. Therefore, it is important to have a reliable and powerful scheme for predicting surface tension^34^, as experimental measurements are not always feasible for all ILs and their mixtures with various substances.

Although there have been some attempts to calculate the surface tension of pure ILs using different methods, there are few studies available in the literature that focus on predicting the surface tension of mixtures containing ILs. Reviews conducted by Tariq et al.^32^ and Gharagheizi et al.^35^ have explored this topic. However, Oliveira et al.^3^ used the Soft Statistical Associating Fluid Theory (soft-SAFT) equation of state and the density gradient theory (DGT) to model the surface tension of mixtures containing [Cnmim][NTf2] ILs with different alkyl chain lengths (n = 1, 2, 5, 6, 8, and 10). A model based on a cubic equation of state and on the geometric similitude concept is proposed by Cardona and Valderrama^36^ to calculate the surface tension of pure substances and mixtures containing organic substances, water, and ILs. The model has been extended to binary and ternary mixtures using simple mixing and combining mixing rules without interaction parameters, so the predictive capabilities of the model are guaranteed. The mixtures are composed of organic solvent + IL and water + ILs. Equations of state (EOS) methods are only applicable to systems for which they have been calibrated. Typically, EOS models rely on adjustable parameters that must be optimized based on experimental data points. Without experimental data and calibrated parameters, these models cannot be fully trusted, and the process of calibration can be time-consuming and complex^37^. Therefore, it is essential to focus on developing and utilizing general models capable of predicting the thermophysical properties of these systems in general, and surface tension in particular.

During recent years, soft computing methods have drawn researchers' attention by virtue of their capability to model and tackle difficult issues that were formerly problematic or impractical to solve^38^. In the field of ILs, several groups around the world have accomplished several studies on the application of the Artificial Neural Networks (ANNs) for prediction the properties of the ILs and their related mixtures such as thermal conductivity of ionic liquids^39^, solubility of supercritical carbon dioxide in ILs^40^, ternary electrical conductivity of IL systems^41^, bubble points of ternary systems involving ILs^42^, viscosity of ternary mixtures containing ILs^43^, binary heat capacity of mixtures containing IL^44^ and melting point of ILs^45^. Also, recommended published papers are^46,47^; for a more applications of different machine learning approaches in the field of ILs.

Various soft computing methods have been employed by researchers to predict the surface tension of pure ILs. For example, Lazzús et al.^48^ utilized a group contribution method based on ANNs to estimate surface tension values of pure ILs, while Atashrouz et al.^49^ developed a mathematical model using Least Square Support Vector Machines (LSSVM) to predict surface tension values of pure ILs. Obaid et al.^50^ used AdaBoost with different base models, including Gaussian Process Regression (GPR), Support Vector Regression (SVR), and Decision Tree (DT) to predict surface tension of different ILs. A review of the current literature reveals that there are only a few studies that have utilized different soft computing techniques to predict surface tension values for binary systems that contain ILs. These methods will be discussed in detail below.

Soleimani and his colleagues^46^ utilized Support Vector Machine (SVM) and LSSVM models combined with Coupled Simulated Annealing (CSA) and Genetic Algorithm (GA) to predict surface tension of binary mixtures consisting of 31 different IL mixtures and 748 data points. The input parameters of their models included temperature, IL properties, and non-IL properties. They found that the CSA-LSSVM model outperformed other models in view of statistical parameters. In another inquiry^51^, they used an ANN model based on the same data points and input parameters. Their model accurately predicted surface tension in terms of statistical analysis. Based on the same dataset and input variables, Setiawan et al.^33^ suggested different ANNs disciplined by four optimization algorithms, namely Teaching–Learning-Based Optimization (TLBO), Particle Swarm Optimization (PSO), GA, and Imperialist Competitive Algorithm (ICA), to estimate surface tension of the binary ILs mixtures. Atashrouz et al.^52^ used GA-LSSVM, GA-SVM, and Group Method of Data Handling Polynomial Neural Network (GMDHPNN) models to estimate surface tension of binary mixtures containing ILs based on 573 data points and 28 different mixtures. Their input data included temperature and properties of ionic and non-ILs. They concluded that GA-LSSVM and GA-SVM models had better prediction ability compared to GMDH-PNN model. Lashkarbolooki^53^ used an ANN model based on 836 data points and 32 different mixtures. The input parameters of the model included temperature, melting temperature, mole fraction, and molecular weight of ionic and non-ILs. Shojaeian and Asadizadeh^54^ proposed an ANN model to predict surface tension of binary mixtures containing ILs based on 1537 data points regarding 33 binary mixtures. In their study, various approaches were developed by utilizing physical properties such as temperature, reduced temperature, critical temperature, critical pressure, critical volume, molecular weight, acentric factor, and critical compressibility factor, along with two distinct mixing rules, as input parameters. In addition, they utilized five different intelligent methods, including Adaptive neuro-fuzzy inference system (ANFIS), ANFIS optimized with Ant Colony Optimization (ANFIS-ACO), ANFIS optimized with Differential Evolution (ANFIS-DE), ANFIS optimized by GA (ANFIS-GA), and ANFIS optimized by PSO (ANFIS-PSO), to predict the surface tension values for the binary mixtures of interest. The results were then compared to those obtained using an ANN model, which was found to have the highest level of accuracy as compared to the other five ANFIS based models. Esmaeili and Hashemipour^55^ used Multi-Gene Genetic Programming (MGGP) to develop correlations for predicting surface tension in binary mixtures containing ILs based on 1414 data related to 37 binary mixtures have been gathered from literature. They presented two correlations for predicting of surface tension of IL and non-IL mixture using just temperature and mole fraction of IL component.

Despite the efforts to create precise models, the review of literature revealed that there is a much larger amount of experimental surface tension data available for binary mixtures containing ILs than what was used in previous studies. Therefore, it is crucial to conduct an in-depth literature search to gather a comprehensive database of experimental surface tension values, which is necessary for developing a comprehensive predictive model.

Over the past few years, Gradient Boosting (GB) Tree model developed by Friedman et al.^56^ has emerged as one of the potent methodologies for predictive data mining. The concept of algorithm for GB Trees rooted in application of boosting method to regression trees. A new version of GB Tree model named stochastic gradient boosting (SGB) tree model, introduced by Friedman^57^, which is appeals to scientific communities and engineers due to enjoys several merits, for instance it works effectively on vast data sets, it is fast, relatively simple, easy to use and requiring the tuning a few parameters. The capability of capturing non-linear associations between inputs and target is one of the main strengths of this improved heuristic model, due to complex inherent structure of real-world data. Also, this promising machine learning scheme is robust to variable outliers, variable collinearity and missing data. Boosted regression tree based models have performed and applied well in various study domains such as carbon dioxide-oil minimum miscibility pressure prediction, carbon dioxide solubility in polymers forecasting^58^, estimation of interfacial tension for geological carbon dioxide storage^59^, predicting carbon dioxide solubility in aqueous amine solutions^60,61^.

As far as we are aware, there is no study on the application of the properties prediction of the surface tension of ILs mixtures using the DT based approaches. Thus, for the first time, this study will present an SGB scheme for predicting binary surface tension values of IL systems using a comprehensive dataset of 4010 experimental surface tension values of binary mixtures containing ILs. Furthermore, we will compare the performance of *SGB* scheme with 18 commonly used computational models. Besides, the effectiveness of each of the input variables on the output of the SGB model, i.e. surface tension, is assessed. Finally, an outlier diagnosis method is employed to examine any ambiguous or inconsistent experimental data.

## Data preparation

All the data assembled (4010 binary surface tension values) for creating the SGB tree model took from the NIST Standard Reference Database^62^, cover temperatures between 278.15 and 348.15 K where the pressure was held constant at atmospheric condition. In total, data points cover 122 distinct binary mixtures comprising 48 different ILs and 20 various non-IL components (water and 19 various organic compounds). The detailed information about binary mixtures, ILs and non-IL constituents presented in the supplementary information (Table S1).

To create the SGB model with satisfactory estimation capabilities of the surface tension for binary mixtures of ILs, some independent variables were taken into account. There are varieties of inter-related factors that affect the surface tension of binary IL mixtures. The relationship that models the interdependency between the surface tension for the binary mixtures and the chosen independent factors based on previous published papers^46,51^, i.e. the temperature ($T$), the mole fraction of the ILs (${x}_{IL}$), molecular weight of IL (${Mw}_{IL}$) and density of IL (${\rho }_{IL}$) together with the boiling point (${Tb}_{non-IL}$) and molecular weight (${Mw}_{non-IL}$) of non-IL component, is expressed as^46,51^:

1 $$\sigma =f\left(T,{ x}_{IL},{MW}_{IL}, {\rho }_{IL},{Tb}_{non-IL}, {Mw}_{non-IL}\right)$$

## Stochastic gradient boosting tree

Stochastic Gradient Boosting (SGB) is a novel branch of traditional Gradient Boosting (GB) developed by Friedman^57^. For enhancing precision and execution speed of the GB with the aim of bettering overall performance^63–65^, SGB merges randomization in the process which is the core principle behind Breiman’s bagging method^66^. Successful applications of this competent method have proven across many domains in the literature^46,58–61,67–74^.

Gradient Boosting (GB) is an ensemble method that transforms weak hypotheses into strong ones by minimizing the loss of the model using a gradient descent-like procedure. GB takes a collection of weak learners, such as decision trees, and adds them to the model to avoid overfitting. Trees are created in a stage-wise fashion, and future weak learners focus more on examples that the previous ones misclassified. The final output of the model is improved by adding the output of the updated tree to the output of the existing sequence of trees.

The training procedure employed in SGB can be examined through the flowchart depicted in Fig. S1, which illustrates that instead of providing all the training instances to a tree, only a fraction of these instances are used for training, selected through sampling without replacement. The sampled data is then utilized for training a tree using only a randomly sampled fraction of the available features for splitting. After a tree is trained, its predictions are made, and the residual errors are computed. These residual errors are multiplied by the learning rate eta ($\eta$) and fed to the next tree in the ensemble. This process is repeated sequentially until all the trees in the ensemble are trained. To predict the output for a new instance in stochastic gradient boosting, a similar procedure is followed as in gradient boosting.

In this study, the SGB algorithms have been executed based on the instructions provided in Friedman’s works^57,63^. Additional information on the mathematical aspects of the SGB model can be found in the literature^57,63,75–77^.

## Results and discussion

### Methodology

The current study utilized the SGB tree model to predict the surface tension of binary mixtures of ILs, as previously mentioned. It is crucial to carefully set the hyper-parameters to ensure the SGB model's maximum generalization ability. Among these parameters, the learning rate (η) has a significant impact on the final outcome. Through an extensive trial and error process, the optimal value for the η was found to be 0.57. The model's performance improves when using a η value of 0.57, as shown in Fig. S2, resulting in a lower Mean Relative Absolute Error (MRAE) value of 0.0039888.

Figure S3 displays the MSE values for the training and test datasets plotted against the number of trees. The initial stages show a rapid leveling off of the error rates. However, as more trees are added, the MSE values for the testing data begin to increase after reaching a minimum error value. This indicates the optimal number of trees to avoid overfitting, as shown by the horizontal green line. The optimal number of trees in this study was determined to be 2976.

### Graphical and statistical evaluation of the SGB model

Various criteria were employed to evaluate the performance accuracy of the SGB tree method. The statistical analysis results were measured in terms of several parameters, including Mean Square Error (MSE), Root Mean Square Error (RMSE), Mean Absolute Error (MAE), Mean Relative Squared Error (MRSE), Mean Relative Absolute Error (MRAE), Relative Absolute Error (RAE), Correlation Coefficient (R), Bias Factor (Bf), and Accuracy Factor (Af). These parameters were calculated using Eqs. (2)–(10) as described in references^51,78^.

2 $$MSE=\frac{1}{N}\sum_{i=1}^{N}{({{y}^{exp}}_{i}-{{y}^{pre}}_{i})}^{2}$$

3 $$RMSE=\sqrt{\frac{1}{N}\sum_{i=1}^{N}{({{y}^{exp}}_{i}-{{y}^{pre}}_{i})}^{2}}$$

4 $$MAE=\frac{1}{N}\sum_{i=1}^{N}\left|{{y}^{exp}}_{i}-{{y}^{pre}}_{i}\right|$$

5 $$MRSE=\frac{1}{N}\sum_{i=1}^{N}{(\frac{{{y}^{exp}}_{i}-{{y}^{pre}}_{i}}{{{y}^{exp}}_{i}})}^{2}$$

6 $$MRAE=\frac{1}{N}\sum_{i=1}^{N}\left|\frac{{{y}^{exp}}_{i}-{{y}^{pre}}_{i}}{{{y}^{exp}}_{i}}\right|$$

7 $$RAE=\sum_{i=1}^{N}\left|\frac{{{y}^{exp}}_{i}-{{y}^{pre}}_{i}}{{{y}^{exp}}_{i}}\right|$$

8 $$R=\left(\left[\sum_{i=1}^{N}({{y}^{exp}}_{i}-\overline{{y }^{exp}})\times ({{y}^{pre}}_{i}-\overline{{y }^{pre}})\right]/\left[\sqrt{\sum_{i=1}^{N}{({{y}^{exp}}_{i}-\overline{{y }^{exp}})}^{2}}\times \sqrt{\sum_{i=1}^{N}{({{y}^{pre}}_{i}-\overline{{y }^{pre}})}^{2}}\right]\right)$$

9 $${B}_{f}={10}^{(\frac{\sum_{i=1}^{N}\mathrm{log}(\frac{{{y}^{pre}}_{i}}{{{y}^{exp}}_{i}})}{N})}$$

10 $${A}_{f}={10}^{(\frac{\sum_{i=1}^{N}\left|\mathrm{log}\frac{{{y}^{pre}}_{i}}{{{y}^{exp}}_{i}}\right|}{N})}$$

where ${y}^{exp}. {y}^{pre}$ and $\overline{y }$ are the experimental value, predicted output and the average value, respectively.

**Figure 1:**
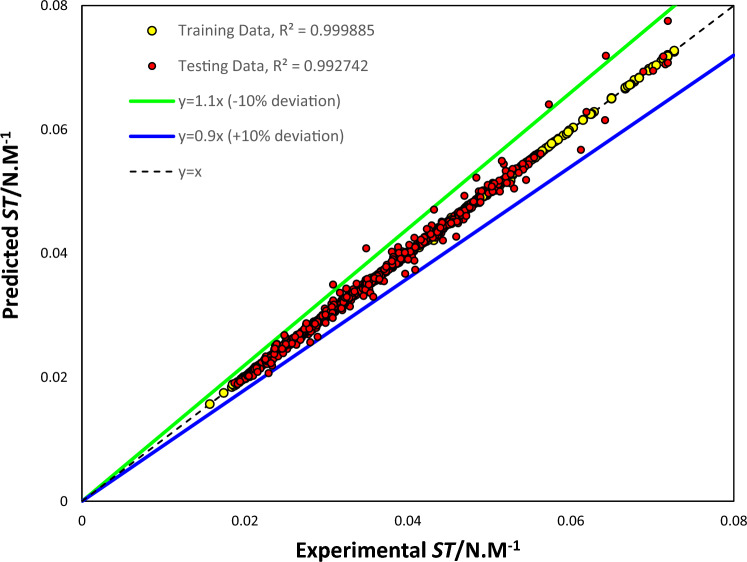
Scatter plot of the SGB tree approach.

Regression plots can be used to validate models, and Fig. 1 in particular shows the regression lines, equations, R-squared values, and 45° line for both the training and test data sets. The R-squared value indicates how well the model outputs and experimental values are related, with an R-squared value of 1 indicating an exact linear relationship and an R-squared value close to zero indicating no linear relationship. The formula for calculating R-squared is given by Eq. (8) squared. It can be seen that the SGB tree estimations have low dispersion, with high R-squared values of 0.99988 and 0.99274 for training and testing, respectively. Equations (11)–(13) are the resulting linear regression equations for the entire dataset, as well as the training and testing subsets.

11 $$y = 1.0009x - 2E-05$$

12 $$y = 0.9997x + 8E-06$$

13 $$y = 1.0056x - 0.0001$$

The SGB model provided highly accurate predictions of the surface tension of binary mixtures, as indicated by the slope value being close to 1 and the intercept having a negligible value.

**Figure 2:**
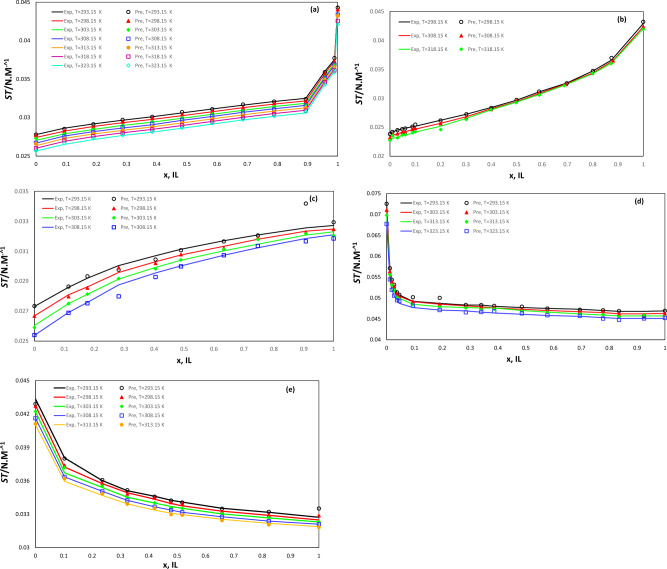
Diagram of surface tension ($\sigma$) of binary mixture (**a**) tributyl phosphate & 1-butyl-3-methylimidazolium hexafluorophosphate, (**b**) butan-1-ol & 1-butyl-3-methylimidazolium L-lactate, (**c**) tetrahydrofuran & 1-butyl-3-methylimidazolium bis(trifluoromethylsulfonyl)imide, (**d**) water & 1-butylpyridinium tetrafluoroborate, and (**e**) dimethyl sulfoxide & 1-butyl-3-methylimidazolium bis(trifluoromethylsulfonyl)imide as a function of temperature (T) and concentration of IL component (x, IL).

Another crucial aspect of creating an accurate predictive model is the model's ability to estimate experimental binary surface tension data accurately, both overestimating and underestimating, across a range of input parameter variations. Figure 2 illustrates the trend plots of SGB predicted values and experimental data points for five selected different binary systems, including tributyl phosphate & 1-butyl-3-methylimidazolium hexafluorophosphate, butan-1-ol & 1-butyl-3-methylimidazolium L-lactate, tetrahydrofuran & 1-butyl-3-methylimidazolium bis(trifluoromethylsulfonyl)imide, water & 1-butylpyridinium tetrafluoroborate, and dimethyl sulfoxide & 1-butyl-3-methylimidazolium bis(trifluoromethylsulfonyl)imide. This figure demonstrates that the developed model can accurately predict the impact of various input parameters on the surface tension of studied binary mixtures. As such, the developed model exhibits an excellent ability to predict the behavior of experimental data over related input parameters. Another observation that can be made from the Fig. 5 is that the surface tension behavior of a mixture consisting of IL changes as the mole fraction of IL varies. For instance, in the tributyl phosphate & 1-butyl-3-methylimidazolium hexafluorophosphate, butan-1-ol & 1-butyl-3-methylimidazolium L-lactate, tetrahydrofuran & 1-butyl-3-methylimidazolium bis(trifluoromethylsulfonyl)imide mixture, the surface tension increases as the mole fraction of IL rises. Conversely, in the water & 1-butylpyridinium tetrafluoroborate mixtures, the surface tension initially decreases with an increase in the mole fraction of IL, but as the concentration of IL continues to rise, the effect of adding more IL becomes less significant.

As mentioned, to ensure that the SGB model can generalize, the collected dataset was divided into two segments: the training set and the test set. The training set was used to fit the SGB model, while the test set provided an unbiased assessment of the model's accuracy. Table 1 presents the key error indexes, including MSE, RMSE, MAE, MRAE, MRSE, R, R^2^, B_f_, and A_f_, for both the training and test subsets of the SGB tree model, as well as for all the data sets. The results in Table 1 indicate that the SGB tree model can accurately predict the surface tension of IL binary mixtures. For example, considering all data points, the B_f_ was obtained 1.0002301 which indicate that the predictions were 0.02301% larger than experimental values, while A_f_ of 1.0039883 means that, on average, the predicted value is 0.39883% different (either smaller or larger) from the experimental value. These results demonstrate the SGB tree model's acceptable accuracy in determining the surface tension of 122 distinct binary mixtures under different conditions. Thus, based on the satisfactory results obtained, it can be concluded that the SGB tree model is a reliable method for predicting the essential physical property of surface tension for binary IL mixtures. Interested readers could refer to the references^78–80^ for detailed discussions of these statistics; in the circumstance of estimation issues; various statistical parameters are as well reviewed in the references^81,82^.

**Table 1:** Calculated values of different errors for the SGB model based on the 4010 collected data.

	All data	Train data	Test data
Root mean square error (RMSE)	0.0003718	0.0001016	0.0008027
Mean absolute error (MAE)	0.0001367	0.0000709	0.0003973
Mean relative absolute error (MRAE)	0.0039888	0.0021908	0.0111031
Mean relative squared error (MRSE)	0.0000891	0.0000096	0.0004034
Correlation coefficient (R)	0.9992264	0.9999423	0.9963646
R-squared (R^2^)	0.9984533	0.9998847	0.9927424
Bias factor (B_f_)	1.0002301	0.9999992	1.0011443
Accuracy factor (A_f_)	1.0039883	1.0021932	1.0111227

**Figure 3:**
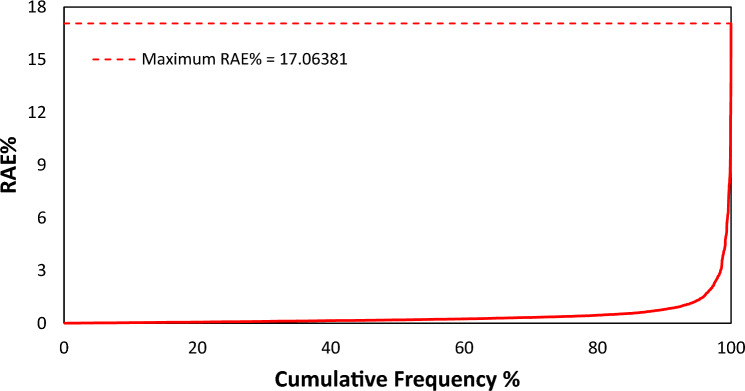
Cumulative frequency versus relative absolute error of the SGB model for predicting surface tension of binary mixtures including ILs.

The cumulative frequency of errors versus RAE% is depicted in Fig. 3. The maximum RAE% value is 17.06, and nearly 92.69% of the data points have errors lower than 1% for predicting surface tension values of binary mixtures containing ILs using the SGB model. In addition, only 4 out of the 4010 data points have errors greater than 10%, which means that 99.90% of the entire dataset has errors less than 10% for the target prediction of interest. This statistical analysis indicates that the SGB tree model is in a satisfactory state and is a precise and reliable tool for predicting the surface tension values of the studied binary mixtures.

### Sensitivity analysis

#### Relative contributions

**Figure 4:**
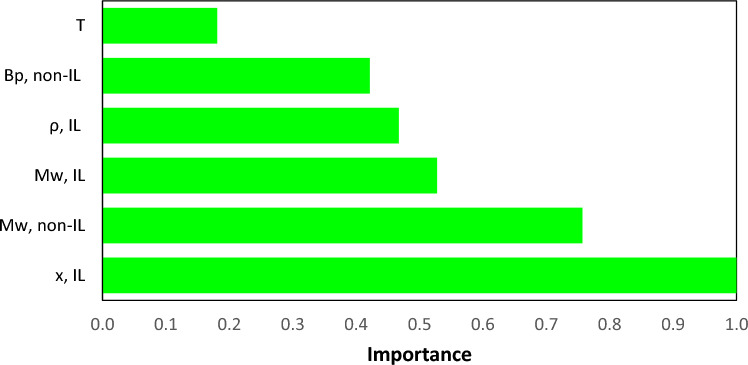
Plot of the importance for each predictor variable for prediction of surface tension of binary mixtures containing ILs.

The SGB algorithm provides the relative influence of each variable on the model’s output, which is a benefit inherent in the decision tree. The variables’ influence is rested on averaging the amount that each variable is decided on for splitting, weighted by the squared improvement to the model as a consequence of each split^83^. Figure 4 illustrates bar graphs that displays the importance scores for each attribute such that the most important variable who have the topmost score assign a value of 1 and then by scaling the others accordingly. Based on the findings presented in Fig. 4, it appears that the SGB model exhibits greater sensitivity to changes in mole fraction (${x}_{IL}$) when predicting surface tension for binary mixtures containing ILs. This observation is consistent with the outcomes reported by Esmaeili and Hashemipour^55^, who utilized the Pearson method to evaluate the efficacy of various parameters in this context. The variables of ${MW}_{non-IL}.{ MW}_{IL}. {\rho }_{IL}. {Tb}_{non-IL} and T$ take the second, third, fourth, fifth and sixth places of sensitivity, respectively.

#### Pearson’s correlation coefficient

In order to conduct a thorough investigation into the surface tension of binary mixtures containing ILs using the SGB model, a sensitivity analysis was performed to determine how input parameters such as $T$, ${x}_{IL}$, ${Mw}_{IL}$, ${\rho }_{IL}$, ${Tb}_{non-IL}$, and ${Mw}_{non-IL}$ affect surface tension. Pearson's correlation coefficient (${r}_{p}$) was used to measure the impact of each parameter on surface tension, with values ranging from − 1 to + 1. A value close to + 1 indicates a strong positive relationship between two variables, with both increasing together, while a value close to − 1 indicates a strong negative relationship with one decreasing as the other increases. A value of 0 indicates no relationship between the variables. The absolute value of the highest ${r}_{p}$ between any input variable and the output variable indicates the most significant influence on the dependent parameter. The following equation was used to calculate the ${r}_{p}$ values:

14 $${r}_{p}=\frac{\sum_{i=1}^{N}({x}_{i}-\overline{x })({y}_{i}-\overline{y })}{\sqrt{\sum_{i=1}^{N}{({x}_{i}-\overline{x })}^{2}\sum_{i=1}^{N}{({y}_{i}-\overline{y })}^{2}}}$$

where ${y}_{i}$, $\overline{y }$ , ${x}_{i}$, and $\overline{x }$ denote the ith output, output average, ith input, and average of input, respectively.

**Figure 5:**
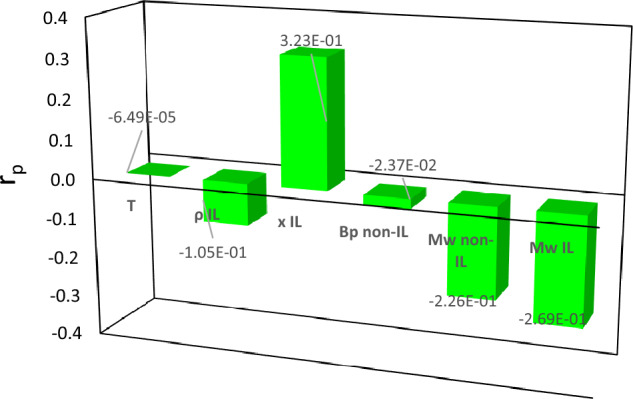
The r_p_ values of input parameters.

The values of ${r}_{p}$ for input parameters for the SGB model are shown in Fig. 5. The results show the negative impacts of $T$, ${Mw}_{IL}$, ${\rho }_{IL}$, ${Tb}_{non-IL}$, and ${Mw}_{non-IL}$ on the surface tension of binary mixtures containing ILs. The ${x}_{IL}$ has the positive and greatest impact on surface tension of binary mixtures with a ${r}_{p}$ of 0.32280 while the variable of T is the least effective parameter with the ${r}_{p}$ of − 0.00006.

### Comparison of the SGB model against the others

Hashemkhani et al.^46^ utilized 748 experimental data points to predict the surface tension of binary mixtures that included ILs using SVM based methods. They conducted a study to optimize the three parameters of the SVM algorithm for predicting surface tension. This was done using a user-defined approach based on prior knowledge and experience. Additionally, GA and CSA algorithms were utilized to find an improved combination of the two hyper parameters embedded in the LSSVM model. The aim was to maximize the generalization performance of the LSSVM model in predicting surface tension. By employing these optimization techniques, the researchers sought to enhance the accuracy and effectiveness of the LSSVM model for surface tension prediction. With the same data set, an ANN^51^ model with a structure containing twelve neurons in it’s both hidden layers and trained by trainbr function was proposed for the purpose of predicting surface tension of binary mixtures. Table 2 demonstrates the computed R and MRAE values for the SGB model, three SVM based models, i.e. SVM, GA-LSSVM, and CSA-LSSVM models and as well as ANN model. Due to higher values of R and lower values of MRAE, the SGB model outperforms the mentioned heuristics approaches in prediction of the surface tension of studied binary mixtures and shows better results. Another point to consider is that the SGB not only generates more accurate outputs, but also covers a more comprehensive data set. It was created based on a large data set of 4010 points, which covers a surface tension range of 0.0157–0.0727 N m^−1^ and temperature range of 278.15–348.15 K. This data set comprises 122 binary systems, with 20 non-IL components and 48 IL components. On the other hand, the ANN, SVM, GA-LSSVM, and CSA-LSSVM were created based on a smaller data set of 748 points, covering 31 binary systems, with 9 non-IL components and 15 IL components. This data set covers a surface tension range of 0.0157–0.07135 N m^−1^ and temperature range of 283.1–348.15 K.

**Table 2:** Evaluation MRAE and R values of different models.

	MRAE	R
SVM^46^	0.037180	0.960176
GA-LSSVM^46^	0.021951	0.977113
CSA-LSSVM^46^	0.013873	0.987044
ANN^51^	0.0042650	0.9995726
SGB	0.0039888	0.9992264

Also, to compare the SGB Model with ANN^53^, SVM^46^, CSA-LSSVM^46^ and GA-LSSVM^46^ models based on 21 different studied binary mixtures that were common in these models, the MRAE in percent was computed for each binary system. It should be mentioned that instead of ${Tb}_{non-IL}$ and ${\rho }_{IL}$, melting point of the IL and non-IL components introduced as model input variables for the proposed ANN model by Lashkarbolooki^53^. He suggested an ANN model for binary surface tension prediction, which comprised one hidden layer with 16 neurons based sing 836 binary surface tension data points obtained within a temperature range of 278.15–348.1 K, and it includes a total of 11 ILs and 11 non-ILs, resulting in 32 binary IL/non-IL systems. The network was trained by trainlm function with 836 collected data points. Table 3 shows obviously the proposed SGB model outperforms the other ones in terms of MRAE%.

**Table 3:** Comparison of the SGB framework with other methods in terms of MRAE% for 21 different binary systems.

		MRAE %				
		ANN^53^	SVM^46^	GA-LSSVM^46^	CSA-LSSVM^46^	SGB
1	1-octene/1-hexyl-3-methylimidazolium bis[(trifluoromethyl)sulfonyl]imide	2.07	3.12	0.98	1.09	0.44
2	Dimethyl Sulfoxide/1-butyl-3-methylimidazolium bis[(trifluoromethyl)sulfonyl]imide	0.34	1.77	1.53	0.62	0.31
3	Dimethyl Sulfoxide/1-ethyl-3-methylimidazolium bis[(trifluoromethyl)sulfonyl]imide	0.34	2.81	0.92	0.20	0.24
4	Acetonitrile/1-butyl-3-methylimidazolium bis[(trifluoromethyl)sulfonyl]imide	0.41	3.32	0.72	0.18	0.31
5	Tetrahydrofuran/1-butyl-3-methylimidazolium bis[(trifluoromethyl)sulfonyl]imide	1.30	2.61	0.23	0.24	0.52
6	Water/1-butyl-3-methylimidazolium tetrafluoroborate	2.62	5.56	5.88	4.05	1.04
7	Water/1-ethyl-3-methylimidazolium tetrafluoroborate	0.87	3.48	3.90	1.70	0.27
8	Ethanol/1-butyl-3-methylimidazolium tetrafluoroborate	0.66	3.80	1.71	0.93	0.31
9	Ethanol/1-hexyl-3-methylimidazolium tetrafluoroborate	1.28	2.06	0.42	0.58	0.27
10	Ethanol/1-methyl-3-octylimidazolium tetrafluoroborate	0.81	1.85	0.45	0.15	1.92
11	Water/1-hexyl-3-methylimidazolium tetrafluoroborate	0.25	2.28	0.13	0.01	0.28
12	Ethanol/1-ethyl-3-methylimidazolium tetrafluoroborate	0.58	12.22	2.60	0.25	0.99
13	Water/1-ethyl-3-methylimidazolium octyl sulfate	2.39	5.73	5.59	4.66	0.88
14	Ethanol/1-ethyl-3-methylimidazolium octyl sulfate	0.23	2.00	0.74	0.14	1.18
15	Water/1-ethyl-3-methylimidazolium ethyl sulfate	1.12	5.94	3.18	1.51	0.30
16	Ethanol/1-ethyl-3-methylimidazolium ethyl sulfate	0.49	1.89	0.20	0.41	1.06
17	1-butanol/1-butyl-3-methylimidazolium bis[(trifluoromethyl)sulfonyl]imide	2.14	2.59	1.33	0.39	1.11
18	1-propanol/1-butyl-3-methylimidazolium bis[(trifluoromethyl)sulfonyl]imide	1.63	2.91	0.81	1.46	0.50
19	Methanol/1-ethyl-3-methylimidazolium methylsulfate	0.96	3.07	0.80	0.34	0.33
20	Ethanol/1-ethyl-3-methylimidazolium methylsulfate	0.55	3.06	1.51	0.46	0.49
21	1-butanol/1-ethyl-3-methylimidazolium methylsulfate	1.32	5.88	5.73	3.04	1.53
	Average	1.03	3.38	1.85	1.07	0.68

Moreover, the computed MRAE% values of three models based on Neural Network (NN) and SVM, viz. GMDH-PNN, GA-SVM and GA-LSSVM which were proposed by Atashrouz et al.^52^ as well as SGB model for 13 different binary mixtures that were common in these models, are tabulated in Table 4. As shown, it is clear that the SGB model presented herein has the smallest MRAE% on average for the common investigated binary mixtures. It is worth noting that in lieu of ${MW}_{IL}. {MW}_{non-IL}.{Tb}_{non-IL}$ and ${\rho }_{IL}$, surface tension of pure components introduced as input variables in Atashrouz et al.^52^ models. It is also worth highlighting that Atashrouz and colleagues^52^ developed two separate models using different datasets; one for ILs mixed with water and another for ILs mixed with organic compounds. In contrast, the SGB model proposed in this study is a unified model that covers both binary systems, including both ILs mixed with water and 19 different organic compounds. This indicates that the SGB model has broader applicability and is more comprehensive than the previous models developed by Atashrouz et al.^52^. Moreover, it should be emphasized that the models proposed by Atashrouz et al.^52^ was constructed using 573 binary surface tension data points that were collected within a temperature range of 283.15–342.8 K, and covering a range of surface tension values from 0.0218 to 0.07160 N M^−1^. The models include 20 ILs and 8 non-ILs, resulting in a total of 28 binary IL/non-IL systems.

**Table 4:** Comparison of MRAE% between GA-LSSVM, GA-SVM, GMDH-PNN and SGB models.

		MRAE%			
		GA-LSSVM^52^	GA-SVM^52^	GMDH-PNN^52^	SGB
1	Dimethyl sulfoxide/1-butyl-3-methylimidazolium bis[(trifluoromethyl)sulfonyl]imide	0.58	0.92	2.45	0.31
2	Dimethyl sulfoxide/1-ethyl-3-methylimidazolium bis[(trifluoromethyl)sulfonyl]imide	0.37	0.68	1.74	0.24
3	Acetonitrile/1-butyl-3-methylimidazolium bis[(trifluoromethyl)sulfonyl]imide	0.56	0.91	2.60	0.31
4	Tetrahydrofuran/1-butyl-3-methylimidazolium bis[(trifluoromethyl)sulfonyl]imide	0.82	0.83	1.30	0.52
5	Ethanol/1-butyl-3-methylimidazolium tetrafluoroborate	2.57	0.88	7.87	0.31
6	Ethanol/1-hexyl-3-methylimidazolium tetrafluoroborate	1.12	0.55	3.10	0.27
7	Ethanol/1-methyl-3-octylimidazolium tetrafluoroborate	3.54	3.48	1.35	1.92
8	Water/1-hexyl-3-methylimidazolium tetrafluoroborate	3.67	5.94	1.48	0.28
9	Water/3-ethyl-1-methylimidazolium butyl sulfate	1.02	0.96	2.26	0.90
10	Ethanol/1-ethyl-3-methylimidazolium octyl sulfate	1.36	1.09	3.30	1.18
11	Ethanol/3-ethyl-1-methyl-1H-imidazolium hexyl sulfate	1.61	1.43	2.76	0.39
12	1-butanol/1-butyl-3-methylimidazolium bis[(trifluoromethyl)sulfonyl]imide	3.27	2.45	8.22	1.11
13	1-propanol/1-butyl-3-methylimidazolium bis[(trifluoromethyl)sulfonyl]imide	0.94	1.56	3.40	0.50
	Average	1.65	1.67	3.22	0.63

In addition, the capability of the SGB model for the purpose of predicting surface tension of mixtures in this study was also compared to the ANN models optimized with GA, PSO, ICA, and TLBO algorithms proposed by Setiawan and colleagues^33^ in terms of R^2^ and MSE values reported in Table 5. As can be seen in Table 5, the SGB model gives better results than PSO-ANN, GA-ANN, ICA-ANN and TLBO-ANN models. The dataset and input parameters utilized in Setiawan et al.’s study^33^ was identical to that in Hashemkhani et al.’s investigation^46^.

**Table 5:** Comparison of TLBO-ANN^33^, PSO-ANN^33^, GA-ANN^33^, ICA-ANN^33^ and SGB models.

	TLBO-ANN	PSO-ANN	GA-ANN	ICA-ANN	SGB
R^2^	0.998	0.996	0.994	0.993	0.998
MSE	0.0000002	0.0000004	0.0000006	0.0000007	0.0000001

Furthermore, a comparison was made between the SGB model and the MGGP model^55^ in terms of their ability to predict the surface tension of 9 binary systems that were present in both models. Table 6, lists the MRAE% values for the both models, and the results suggest that the surface tension predictions by the proposed SGB model have better agreement with the experimental data compared to MGGP model. It should be noted that, the MGGP model was developed using a data set containing 1414 data points, which pertains to 37 binary systems and includes 10 non-IL components and 20 IL components. This data set covers a temperature range spanning from 278.15 to 348.15 K.

**Table 6:** Comparison of MGGP^55^ and SGB models in terms of MRAE%.

Binary System	MRAE%	
	MGGP	SGB
1-octene/1-hexyl-3-methylimidazolium bis[(trifluoromethyl)sulfonyl]imide	2.813	0.438
Dimethyl Sulfoxide/1-butyl-3-methylimidazolium bis[(trifluoromethyl)sulfonyl]imide	1.309	0.308
Dimethyl Sulfoxide/1-ethyl-3-methylimidazolium bis[(trifluoromethyl)sulfonyl]imide	0.940	0.245
Acetonitrile/1-butyl-3-methylimidazolium bis[(trifluoromethyl)sulfonyl]imide	0.791	0.306
Tetrahydrofuran/1-butyl-3-methylimidazolium bis[(trifluoromethyl)sulfonyl]imide	0.440	0.523
Methanol/1-butyl-3-methylimidazolium L-lactate	1.804	0.569
Water/1-butyl-3-methylimidazolium L-lactate	0.824	0.536
1-butanol/1-butyl-3-methylimidazolium L-lactate	0.996	0.357
Ethanol/1-butyl-3-methylimidazolium L-lactate	0.689	0.718
Average	1.178	0.444

Finally, Table 7 presents a comparison of the MSE values of six models developed by Shojaeian and Asadizadeh^54^, including ANFIS, ANFIS-ACO, ANFIS-DE, ANFIS-GA, ANFIS-PSO, and ANN, with the SGB model. The authors used 1537 data points from 33 binary mixtures comprising 15 unique IL components and 11 individual non-IL substances to predict surface tension across a temperature range of 278.15–338.15 K, with a surface tension range of 0.0189–0.0727 N M^−1^. To prepare the input parameters, they used physical properties such as temperature, reduced temperature, critical temperature, critical pressure, critical volume, molecular weight, acentric factor, and critical compressibility factor, as well as two different mixing rules. The ANN models proposed by Shojaeian and Asadizadeh had one hidden layer with 10 neurons and used the training function trainlm. In the ANFIS-based models, ACO, DE, GA, and PSO algorithms were introduced to obtain the optimum parameters. Table 7 shows that the SGB model is more accurate and superior to both the ANN model and the five ANFIS-based models proposed by Shojaeian and Asadizadeh^54^.

**Table 7:** Comparison of ANFIS^54^, ANFIS-ACO^54^, ANFIS-DE^54^, ANFIS-GA^54^, ANFIS-PSO^54^, ANN^54^ and SGB models.

	ANFIS	ANFIS-ACO	ANFIS-DE	ANFIS-GA	ANFIS-PSO	ANN	SGB
MSE	0.000811	0.0167	0.0163	0.00507	0.00421	0.0000620	0.0000001

### Outlier detection

The detection of outliers is crucial in the development of mathematical models^84^. Outliers refer to observations that deviate from the bulk of data obtained under the same conditions^84,85^. It is common to encounter outliers or doubtful data in projects involving data collection, and this is especially true for large datasets like the one used in this study. In addition to errors in experimental measurements, data entry errors can also contribute to the presence of outliers, particularly when data is recorded manually^86^. To develop reliable predictive models, it is essential to have accurate data points from experimental tests^87^. However, even if the data is obtained from reputable sources, errors in experimental measurements may affect the model's prediction capability. Removing potential outliers can enhance model performance, but this requires a novel technique to identify them. The Leverage approach is used in this study to assess the quality of experimental data points and determine the best model's range of applicability.

The leverage approach involves the use of a hat matrix (H) to calculate the hat indices or leverage of data points as follows^84,85,88,89^:

15 $$H=X{({X}^{t}X)}^{-1}{X}^{t}$$

The equation given uses a two-dimensional matrix X with N rows (representing the data points) and k columns (representing the model parameters), along with a transpose multiplier t. The hat values of data are represented by the diagonal components of the H matrix, which are obtained using Eq. (15). These H values are then used in a Williams plot to visually identify outlier and suspected data points, as well as to determine the correlation between the H indices and standardized residuals. A Williams plot is essentially a graph that plots standardized residuals against hat values and can be used to differentiate valid data, suspected data, and out-of-leverage data. The standardized residuals (SR), also known as cross-validation residuals, are calculated for each data point using the following formula^89^:

16 $${SR}_{i}=\frac{{{y}^{exp}}_{i}-{{y}^{pre}}_{i}}{RMSE\sqrt{(1-{H}_{ii})}}$$

The hat index of the ith data point is denoted by Hii in the equation given above.

**Figure 6:**
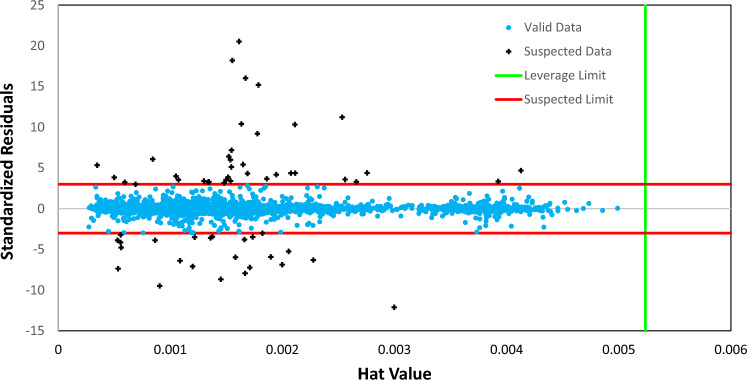
The Williams plot of SGB model for predicting surface tension of binary mixtures containing ILs.

The Leverage approach utilizes a warning leverage parameter (${\mathrm{H}}^{*}$) for accepting or rejecting model outputs and measurements. This parameter is determined using the equation H = 3(k + 1)/N. Typically, a leverage value of 3 is used as the threshold, indicating that acceptable data should be within the range of − 3 to + 3 standard deviations from the mean. These bounds are illustrated by two red lines in Fig. 6. If the majority of data points fall within the ranges of $0 \le {H}_{ii}\le {\mathrm{H}}^{*}$ and $-3 \le {SR}_{i} \le 3$, it can be concluded that the model and its predictions are valid and reliable, and that the experimental data used for developing the model are also reliable and valid^84,89^.

Based on Fig. 6, it can be seen only a small portion (1.5%) of the data points were flagged as suspected. So, it can be inferred that the proposed model is highly applicable, reliable, accurate, and statistically valid, as the majority of the data points fall within the specified ranges of H and R.

## Conclusion

The capability of the *SGB* tree model in handling 122 different types of binary systems, in predicting of surface tension of binary mixtures containing ILs based on a comprehensive data set of 4010 experimental data points consists of 48 different ILs and 20 various non-IL components, was examined. In the *SGB* tree model, the system conditions of temperature and IL component composition as well as molecular weight of IL and non-IL components, density of IL component and normal boiling point of non-IL component are used as input variables. It is notable that SGB tree model has been used for the first time for prediction/estimation of properties of mixtures especially those containing IL. Based on the results presented, the main contributions of the current research include:

1. Experimental surface tensions of studied binary systems show a consistency and good agreement with results of SGB tree model.
2. The MRAE and R values of the SGB models for predicting of mixtures containing ILS were nearly 0.003989 and 0.99923 respectively.
3. The comparison between the results of 18 various computational approaches reveals that the *SGB* method is visibly superior to the SVM, GA-SVM, GA-LSSVM, CSA-LSSVM, GMDH-PNN, three based ANNs, PSO-ANN, GA-ANN, ICA-ANN, TLBO-ANN, ANFIS, ANFIS-ACO, ANFIS-DE, ANFIS-GA, ANFIS-PSO, and MGGP models in the respect of accuracy.
4. Furthermore, with the bar graph of the predictor importance, the mole fraction of IL component was recognized as the variable that makes the major contributions to the prediction of the dependent variable of interest.
5. The Leverage mathematical algorithm was employed to detect outliers and assess the applicability domain of the SGB model proposed in this study. The analysis revealed that a very small percentage, specifically 1.5%, of the overall dataset was deemed questionable and did not meet the expected criteria.
6. In addition to the high accuracy of the predicted surface tensions, the most important advantage of the model of binary surface tensions proposed in this study, is that the proposed SGB tree model constructed exclusively based on experimental data which makes it attractive for scientists and engineers to apply such ensemble learning tool for rough estimation of the surface tension of any desired binary mixtures comprised of ILs.
7. The findings of this study can be used in industries that use ILs, particularly in the design and optimization of new processes on an industrial scale.
8. Due to the largest available dataset was applied, a dependable technique was put forth to predict the surface tension of numerous binary mixtures containing various ILs. Nevertheless, it has a limitation: although the SGB method is broadly applicable, its predictive ability is confined to binary systems that closely resemble those used to create the model. It is not advisable to apply the developed tool to binary systems that are entirely dissimilar from the ones studied, though it may provide a rough approximation of the surface tension of such mixtures.
9. Future directions of this work could involve applying the developed models to predict the surface tension of new binary mixtures containing different ILs such as phosphonium and sulfonium based-ILs and evaluating their performance against experimental data. Additionally, the developed model could be used in process optimization and design for various industrial applications. Further research could also investigate the feasibility of applying these models to ternary and multicomponent systems containing ILs. More research could also investigate the feasibility of applying this model to other types of properties of mixtures containing ILs.

### Supplementary Information

Supplementary Information.

## Data Availability

All data generated or analyzed during this study are included in this published article.

## Abbreviations

ANFIS: Adaptive neuro-fuzzy inference system
ANN: Artificial neural network
ACO: Ant colony optimization CSA coupled simulated annealing
DE: Differential evolution
DT: Decision tree
DGT: Density gradient theory
EOR: Enhanced oil recovery
GA: Genetic algorithm
GB: Gradient boosting
GMDH-PNN: Group method of data handling polynomial neural network
GPR: Gaussian process regression
ICA: Imperialist competitive algorithm
IFT: Interfacial tension
IL: Ionic liquid
LSSVM: Least square support vector machine
MAE: Mean absolute error
MGGP: Multi-gene genetic programming
MRAE: Mean relative absolute error
MRSE: Mean relative squared error
MSE: Mean square error
NIST: National institute of standards and technology
NN: Neural network
PSO: Particle swarm optimization
RAE: Relative absolute error
Soft-SAFT: Soft statistical associating fluid theory
SGB: Stochastic gradient boosting
SR: Standardized residuals
SVM: Support vector machine
SVR: Support vector regression
TLBO: Teaching–learning-based optimization

## List of symbols

A_f_: Accuracy factor
B_f_: Bias factor
H: Hat value
H^*^: Warning leverage
k: Number of input parameters
R: Correlation coefficient
T: Temperature
t: Transpose multiplier
${x}_{IL}$: IL component composition
${\mathrm{Mw}}_{\mathrm{IL}}$: Molecular weight of IL components
${\rho }_{IL}$: Density of IL components
${r}_{p}$: Pearson’s correlation coefficient
${Tb}_{non-IL}$: Boiling point non-IL component
${Mw}_{non-IL}$: Molecular weight of non-IL component
$$\sigma$$: Surface tension
$$\eta$$: Learning rate
N: Total number of data points
$${x}_{i}$$: Ith input
$\overline{x }$: Average of input
${{y}^{exp}}_{i}$: Experimental output at the sampling point $i$
${{y}^{pre}}_{i}$: *i*Th output of the model
$\overline{y }$: Output average of output
