# Supplementary material for: Insights into the estimation of surface tensions of mixtures based on designable green materials using an ensemble learning scheme
Source: Sci Rep. 2023 Aug 29;13:14145. doi: 10.1038/s41598-023-41448-z (PMC10465615; doi:10.1038/s41598-023-41448-z)
Supplement: Supplementary file 1 — Supplementary Information. [file 41598_2023_41448_MOESM1_ESM.docx]

**Supplementary Information**

**Insights into the Estimation of Surface Tensions of Mixtures based on Designable Green Materials Using an Ensemble Learning Scheme**

Reza Soleimani ^1^, Amir Hossein Saeedi Dehaghani ^2,*^

*^1^ Department of Chemical Engineering, Faculty of Chemical Engineering, Tarbiat Modares University, P.O. Box 14115-143, Tehran, Iran*

*^2^ Department of Petroleum Engineering, Faculty of Chemical Engineering, Tarbiat Modares University, P.O. Box 14115-143, Tehran, Iran*

(*Corresponding author’s e-mail: asaeedi@modares.ac.ir)

| **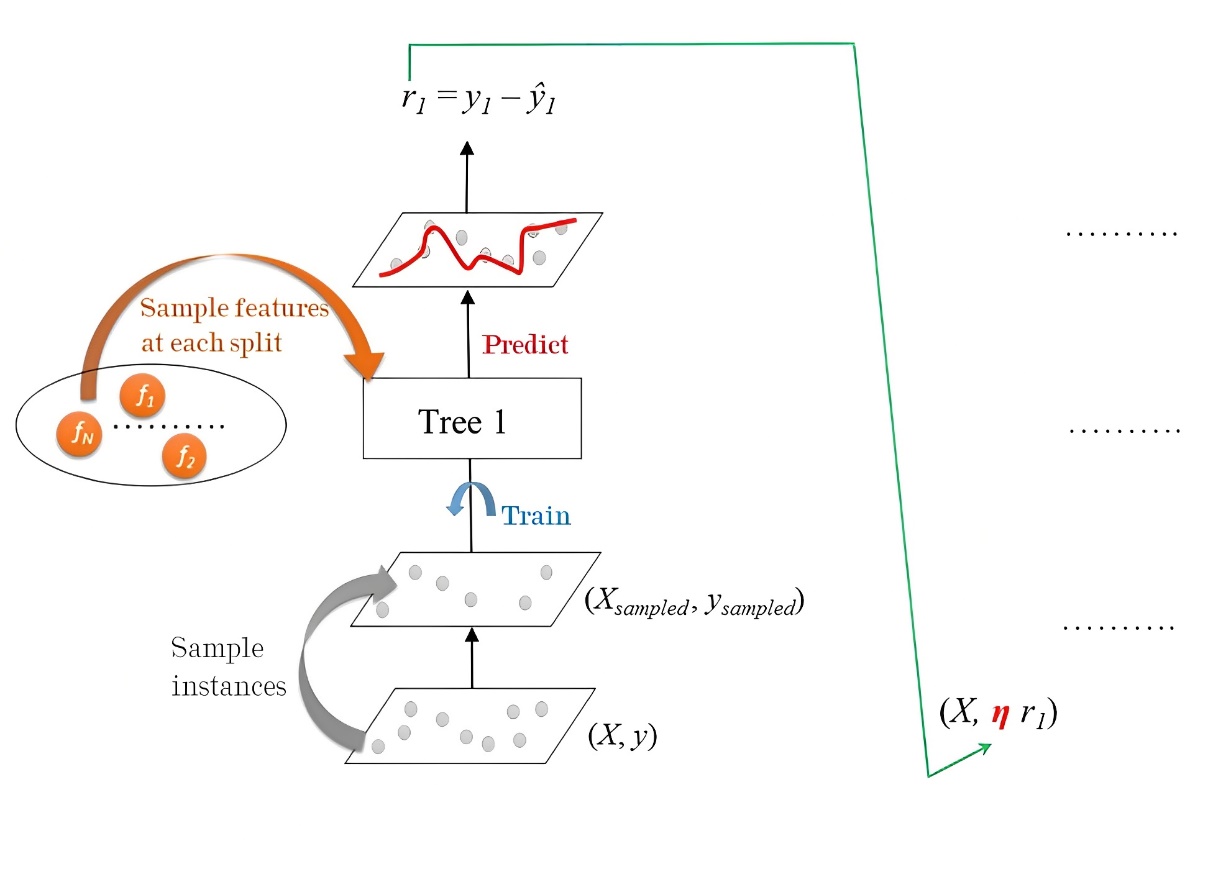** |
| --- |
| **Figure S1**. Flowchart of SGB training procedure. |

|  |
| --- |
| **Figure S2**. Effect of learning rate on performance of the SGB model in terms of MRAE% value. |

|  |
| --- |
| **Figure S3.** Graph of the *MSE* over the successive boosting steps for the training data and the testing samples for estimation of surface tensions of binary mixtures containing ILs. |

**Table S1.** Ranges of temperatures, specific density, and surface tensions of used ILs and binary systems in this study^1^.

| No | Binary System | Data Point | Method | ΔT  K | IL Formula | Mw, IL g.mol^-1^ | Δρ IL  kg.m^-3^ | Reference Δρ values | Δσ^Liq^  N.m^-1^ | Reference Δσ^Liq^ values |
| --- | --- | --- | --- | --- | --- | --- | --- | --- | --- | --- |
| 1 | tributyl phosphate / 1-butyl-3-methylimidazolium hexafluorophosphate | 91 | Plate method | 293.15 - 323.15 | C_8_H_15_F_6_N_2_P | 284.19 | 1347.5 - 1372.3 | ^2^ | 0.02566 - 0.04456 | ^3^ |
| 2 | tributyl phosphate / 1-butyl-3-methylimidazolium tetrafluoroborate | 91 | Plate method | 293.15 - 323.15 | C_8_H_15_BF_4_N_2_ | 226.03 | 1184.9 -1206 | ^2^ | 0.02566 - 0.04475 | ^3^ |
| 3 | tributyl phosphate / 1-butyl-3-methylimidazolium bis(trifluoromethylsulfonyl)imide | 91 | Plate method | 293.15 - 323.15 | C_10_H_15_F_6_N_3_O_4_S_2_ | 419.36 | 1413.1- 1441.6 | ^2^ | 0.02566 - 0.03329 | ^3^ |
| 4 | ethanol / 1-ethyl-3-methylimidazolium acetate | 77 | Plate Method | 278.15 -338.15 | C_8_H_14_N_2_O_2_ | 170.21 | 1075 - 1112.4 | ^4^ | 0.0189 - 0.0481 | ^5^ |
| 5 | propan-1-ol / 1-ethyl-3-methylimidazolium acetate | 77 | Wilhelmy plates | 288.15 - 348.15 | C_8_H_14_N_2_O_2_ | 170.21 | 1069.2 - 1105.6 | ^4^ | 0.019 - 0.0477 | ^6^ |
| 6 | ethanol / 1-ethyl-3-(2-methoxyethyl)imidazolium trifluoromethanesulfonate | 77 | Maximum bubble pressure | 288.15 - 318.15 | C_9_H_15_F_3_N_2_O_4_S | 304.28 | 1323.6 - 1348.9 | ^7^ | 0.019 - 0.0363 | ^7^ |
| 7 | propan-1-ol / 1-ethyl-3-(2-methoxyethyl)imidazolium trifluoromethanesulfonate | 77 | Maximum bubble pressure | 288.15 - 318.15 | C_9_H_15_F_3_N_2_O_4_S | 304.28 | 1323.6 - 1348.9 | ^7^ | 0.0206 - 0.0363 | ^7^ |
| 8 | propan-2-ol / 1-ethyl-3-(2-methoxyethyl)imidazolium trifluoromethanesulfonate | 77 | Maximum bubble pressure | 288.15 - 318.15 | C_9_H_15_F_3_N_2_O_4_S | 304.28 | 1323.6 - 1348.9 | ^7^ | 0.0184 - 0.0363 | ^7^ |
| 9 | acetonitrile / 1-(2-methoxyethyl)-1-methylpyrrolidinium bis((trifluoromethyl)sulfonyl)amide | 77 | Maximum bubble pressure | 288.15 - 318.15 | C_10_H_18_F_6_N_2_O_5_S_2_ | 424.37 | 1435.9 - 1464 | ^8^ | 0.0266 - 0.0369 | ^9^ |
| 10 | acetonitrile / 1-allyl-3-methyl-1H-imidazolium bis((trifluoromethyl)sulfonyl)amide | 77 | Maximum bubble pressure | 288.15 - 318.15 | C_9_H_11_F_6_N_3_O_4_S_2_ | 403.31 | 1479.5 - 1510 | ^10^ | 0.0266 - 0.0352 | ^9^ |
| 11 | butan-1-ol / 1-ethyl-3-(2-methoxyethyl)imidazolium thiocyanate | 77 | Maximum bubble pressure | 288.15 - 318.15 | C_9_H_15_N_3_OS | 213.3 | 1120.1 - 1139.5 | ^11^ | 0.021 - 0.0514 | ^11^ |
| 12 | propan-2-ol / 1-ethyl-3-(2-methoxyethyl)imidazolium thiocyanate | 77 | Maximum bubble pressure | 288.15 - 318.15 | C_9_H_15_N_3_OS | 213.3 | 1120.1 - 1139.5 | ^11^ | 0.0191 - 0.0514 | ^11^ |
| 13 | ethanol / 1-ethyl-3-(2-methoxyethyl)imidazolium thiocyanate | 77 | Maximum bubble pressure | 288.15 - 318.15 | C_9_H_15_N_3_OS | 213.3 | 1120.1 - 1139.5 | ^11^ | 0.02 - 0.0514 | ^11^ |
| 14 | propan-1-ol / 1-ethyl-3-(2-methoxyethyl)imidazolium thiocyanate | 77 | Maximum bubble pressure | 288.15 - 318.15 | C_9_H_15_N_3_OS | 213.3 | 1120.1 - 1139.5 | ^11^ | 0.0209 - 0.0514 | ^11^ |
| 15 | ethanol / 1-butylpyridinium dicyanamide | 77 | Maximum bubble pressure | 288.15 - 318.15 | C_11_H_14_N_4_ | 202.26 | 1057 - 1075 | ^12^ | 0.02 - 0.0563 | ^13^ |
| 16 | propan-1-ol / 1-butylpyridinium dicyanamide | 77 | Maximum bubble pressure | 288.15 - 318.15 | C_11_H_14_N_5_ | 203.26 | 1057 - 1075 | ^12^ | 0.0216 - 0.0563 | ^13^ |
| 17 | propan-2-ol / 1-butylpyridinium dicyanamide | 77 | Maximum bubble pressure | 288.15 - 318.15 | C_11_H_14_N_6_ | 204.26 | 1057 - 1075 | ^12^ | 0.0194 - 0.0563 | ^13^ |
| 18 | methanol / 1-(2-methoxyethyl)-3-methyl-1H-imidazolium bis((trifluoromethyl)sulfonyl)amide | 77 | Maximum bubble pressure | 288.15 - 318.15 | C_9_H_13_F_6_N_3_O_5_S_2_ | 421.33 | 1484.7 - 1515.3 | ^13^ | 0.0205 - 0.0414 | ^13^ |
| 19 | ethanol / 1-(2-methoxyethyl)-3-methyl-1H-imidazolium bis((trifluoromethyl)sulfonyl)amide | 77 | Maximum bubble pressure | 288.15 - 318.15 | C_9_H_13_F_6_N_3_O_5_S_2_ | 421.33 | 1484.7 - 1515.3 | ^13^ | 0.02 - 0.0414 | ^13^ |
| 20 | water / 1-butylpyridinium tetrafluoroborate | 72 | Drop volume | 293.15 - 323.15 | C_9_H_14_BF_4_N | 223.02 | 1196.68 - 1217.11 | ^14^ | 0.0451 - 0.0727 | ^15^ |
| 21 | propan-2-ol / 1-ethyl-3-methylimidazolium acetate | 66 | Wilhelmy plates | 288.15 - 338.15 | C_8_H_14_N_2_O_2_ | 170.21 | 1075 - 1105.6 | ^4^ | 0.0174 - 0.0477 | ^6^ |
| 22 | water / 1-propylpyridinium tetrafluoroborate | 64 | Drop volume | 293.15 - 323.15 | C_8_H_12_BF_4_N | 208.99 | 1235.3 - 1256.6 | ^16^ | 0.0485 - 0.0727 | ^15^ |
| 23 | methanol / 1-butylpyridinium tetrafluoroborate | 56 | Drop volume | 293.15 - 323.15 | C_9_H_14_BF_4_N | 223.02 | 1196.68 - 1217.11 | ^14^ | 0.02005 - 0.0469 | ^17^ |
| 24 | methanol / 1-butyl-3-methylpyridinium tetrafluoroborate | 56 | Drop volume | 293.15 - 323.15 | C_10_H_16_BF_4_N | 237.05 | 1165.1  - 1186 | ^18^ | 0.02005 - 0.04521 | ^17^ |
| 25 | ethanol / 1-butyl-4-methylpyridinium tetrafluoroborate | 56 | Drop volume | 293.15 - 323.15 | C_10_H_16_BF_4_N | 237.05 | 1165.5 - 1186.1 | ^18^ | 0.01984 - 0.04582 | ^17^ |
| 26 | methanol / 1-butyl-4-methylpyridinium tetrafluoroborate | 56 | Drop volume | 293.15 - 323.15 | C_10_H_16_BF_4_N | 237.05 | 1165.5 - 1186.1 | ^18^ | 0.02005 - 0.04582 | ^17^ |
| 27 | ethanol / 1-butyl-3-methylpyridinium tetrafluoroborate | 56 | Drop volume | 293.15 - 323.15 | C_10_H_16_BF_4_N | 237.05 | 1165.1  - 1186 | ^18^ | 0.01984 - 0.04521 | ^17^ |
| 28 | methanol / 1-ethyl-3-methylimidazolium acetate | 55 | Wilhelmy plate | 278.2 - 318.2 | C_8_H_14_N_2_O_2_ | 170.21 | 1087 - 1112.4 | ^4^ | 0.021 - 0.0481 | ^6^ |
| 29 | water / 1-ethyl-3-methylimidazolium tetrafluoroborate | 55 | Ring tensiometer | 298.15 - 338.15 | C_6_H_11_BF_4_N_2_ | 197.97 | 1253.3 - 1283.3 | ^19^ | 0.05045 - 0.0718 | ^20^ |
| 30 | water / 1-butyl-3-methylimidazolium tetrafluoroborate | 55 | Ring tensiometer | 298.15 - 338.15 | C_8_H_15_BF_4_N_2_ | 226.03 | 1174.4  - 1202.5 | ^2^ | 0.04155 - 0.0718 | ^20^ |
| 31 | methanol / 1-propylpyridinium tetrafluoroborate | 52 | Drop volume | 293.15 - 323.15 | C_8_H_12_BF_4_N | 208.99 | 1235.3 - 1256.6 | ^16^ | 0.02005 - 0.05129 | ^17^ |
| 32 | dimethyl sulfoxide / 1-butyl-3-methylimidazolium bis(trifluoromethylsulfonyl)imide | 50 | Pendant drop shape | 293.15 - 313.15 | C_10_H_15_F_6_N_3_O_4_S_2_ | 419.36 | 1422.5 - 1441.6 | ^2^ | 0.03188 - 0.04336 | ^21^ |
| 33 | methanol / 1-butyl-3-methylimidazolium L-lactate | 48 | Wilhelmy plate | 298.15 - 318.15 | C_11_H_20_N_2_O_3_ | 228.29 | 1097.7 - 1111.2 | ^22^ | 0.02046 - 0.04292 | ^22^ |
| 34 | water / 1-butyl-3-methylimidazolium L-lactate | 48 | Wilhelmy plate | 298.15 - 318.15 | C_11_H_20_N_2_O_3_ | 228.29 | 1097.7 - 1111.2 | ^22^ | 0.04182 - 0.07185 | ^22^ |
| 35 | butan-1-ol / 1-butyl-3-methylimidazolium L-lactate | 48 | Wilhelmy plate | 298.15 - 318.15 | C_11_H_20_N_2_O_3_ | 228.29 | 1097.7 - 1111.2 | ^22^ | 0.02262 - 0.04292 | ^22^ |
| 36 | pentan-1-ol / 1-butyl-3-methylimidazolium thiocyanate | 45 | Ring tensiometer | 298.15 - 338.15 | C_9_H_15_N_3_S | 197.3 | 1046.1 - 1069.5 | ^19^ | 0.02131 - 0.0426 | ^23^ |
| 37 | butan-1-ol / 1-butyl-3-methylimidazolium thiocyanate | 45 | Ring tensiometer | 298.15 - 338.15 | C_9_H_15_N_3_S | 197.3 | 1046.1 - 1069.5 | ^19^ | 0.02034 - 0.0474 | ^23^ |
| 38 | dimethyl sulfoxide / 1-ethyl-3-methylimidazolium bis((trifluoromethyl)sulfonyl)imide | 45 | Pendant drop shape | 293.15 - 313.15 | C_8_H_11_F_6_N_3_O_4_S_2_ | 391.3 | 1503.38 - 1523.53 | ^24^ | 0.0351 - 0.04336 | ^21^ |
| 39 | acetonitrile / 1-butyl-3-methylimidazolium bis(trifluoromethylsulfonyl)imide | 45 | Pendant drop shape | 293.15 - 313.15 | C_10_H_15_F_6_N_3_O_4_S_2_ | 419.36 | 1422.5 - 1441.6 | ^2^ | 0.02643 - 0.03278 | ^21^ |
| 40 | ethanol / 1-butyl-3-methylimidazolium L-lactate | 45 | Wilhelmy plate | 298.15 - 318.15 | C_11_H_20_N_2_O_3_ | 228.29 | 1097.7 - 1111.2 | ^22^ | 0.02048 - 0.04292 | ^22^ |
| 41 | water / 1-hexyl-3-methylimidazolium tetrafluoroborate | 45 | Ring tensiometer | 298.15 - 338.15 | C_10_H_19_BF_4_N_2_ | 254.08 | 1117.69 - 1145.11 | ^25^ | 0.03452 - 0.0718 | ^20^ |
| 42 | hexan-1-ol / 1-butyl-3-methylimidazolium thiocyanate | 40 | Ring tensiometer | 298.15 - 338.15 | C_9_H_15_N_3_S | 197.3 | 1046.1 - 1069.5 | ^19^ | 0.02223 - 0.0389 | ^23^ |
| 43 | tetrahydrofuran / 1-butyl-3-methylimidazolium bis(trifluoromethylsulfonyl)imide | 40 | Pendant drop shape | 293.15 - 308.15 | C_10_H_15_F_6_N_3_O_4_S_2_ | 419.36 | 1427.2 - 1441.6 | ^2^ | 0.02538 - 0.03272 | ^21^ |
| 44 | ethanol / 1-methyl-3-octylimidazolium bis(trifluoromethylsulfonyl)imide | 39 | Wilhelmy plate | 283.15 - 313.15 | C_14_H_23_F_6_N_3_O_4_S_2_ | 475.47 | 1307 - 1331 | ^26^ | 0.02293 - 0.03196 | ^27^ |
| 45 | ethanol / 1-butylpyridinium tetrafluoroborate | 39 | Drop volume | 303.15 - 323.15 | C_9_H_14_BF_4_N | 223.02 | 1196.68 - 1210.29 | ^14^ | 0.01984 - 0.0463 | ^17^ |
| 46 | ethanol / 1-butyl-3-methylimidazolium bis(trifluoromethylsulfonyl)imide | 36 | Wilhelmy plate | 283.15 - 313.15 | C_10_H_15_F_6_N_3_O_4_S_2_ | 419.36 | 1422.5 - 1451.3 | ^2^ | 0.02266 - 0.03342 | ^27^ |
| 47 | propan-1-ol / 1-methyl-3-octylimidazolium bis(trifluoromethylsulfonyl)imide | 33 | Wilhelmy plate | 283.15 - 313.15 | C_14_H_23_F_6_N_3_O_4_S_2_ | 475.47 | 1307 - 1331 | ^26^ | 0.02328 - 0.03172 | ^27^ |
| 48 | propan-2-ol / 1-butyl-3-methylimidazolium bis(trifluoromethylsulfonyl)imide | 33 | Wilhelmy plate | 283.15 - 313.15 | C_10_H_15_F_6_N_3_O_4_S_2_ | 419.36 | 1422.5 - 1451.3 | ^2^ | 0.02077 - 0.03334 | ^27^ |
| 49 | propan-2-ol / 1-methyl-3-octylimidazolium bis(trifluoromethylsulfonyl)imide | 30 | Wilhelmy plate | 283.15 - 313.15 | C_14_H_23_F_6_N_3_O_4_S_2_ | 475.47 | 1307 - 1331 | ^26^ | 0.02125 - 0.03152 | ^27^ |
| 50 | propan-1-ol / 1-butyl-3-methylimidazolium bis(trifluoromethylsulfonyl)imide | 30 | Wilhelmy plate | 283.15 - 313.15 | C_10_H_15_F_6_N_3_O_4_S_2_ | 419.36 | 1422.5 - 1451.3 | ^2^ | 0.02311 - 0.03323 | ^27^ |
| 51 | methanol / 1-butyl-1-methylpyrrolidinium bis[(trifluoromethyl)sulfonyl]imide | 30 | Pendant drop shape | 288.15 - 308.15 | C_11_H_20_F_6_N_2_O_4_S_2_ | 422.4 | 1386.1 - 1403.8 | ^8^ | 0.02101 - 0.03371 | ^28^ |
| 52 | water / 1-butyl-3-methylimidazolium tetrafluoroborate | 30 | Drop volume | 298.15 | C_8_H_15_BF_4_N_2_ | 226.03 | 1202.5 | ^2^ | 0.04533 - 0.07135 | ^29^ |
| 53 | benzo[b]thiophene / 1-butyl-1-methylpyrrolidinium tricyanomethanide | 28 | Ring tensiometer | 308.15 - 338.15 | C_13_H_20_N_4_ | 232.33 | 982.44  - 1000.55 | ^30^ | 0.03449 - 0.04804 | ^31^ |
| 54 | butan-1-ol / 1-methyl-3-octylimidazolium bis(trifluoromethylsulfonyl)imide | 27 | Wilhelmy plate | 283.15 - 313.15 | C_14_H_23_F_6_N_3_O_4_S_2_ | 475.47 | 1307 - 1331 | ^26^ | 0.0237 - 0.03123 | ^27^ |
| 55 | pentan-1-ol / 1-methyl-3-octylimidazolium bis(trifluoromethylsulfonyl)imide | 27 | Wilhelmy plate | 283.15 - 313.15 | C_14_H_23_F_6_N_3_O_4_S_2_ | 475.47 | 1307 - 1331 | ^26^ | 0.02428 - 0.03101 | ^27^ |
| 56 | acetonitrile / 1-butyl-1-methylpyrrolidinium bis[(trifluoromethyl)sulfonyl]imide | 27 | Pendant drop shape | 288.15 - 308.15 | C_11_H_20_F_6_N_2_O_4_S_2_ | 422.4 | 1386.1 - 1403.8 | ^8^ | 0.02715 - 0.03371 | ^28^ |
| 57 | water / 3-ethyl-1-methylimidazolium butyl sulfate | 27 | Drop volume | 298.15 | C_10_H_20_N_2_O_4_S | 264.34 | 1175.7 | ^32^ | 0.03908 - 0.04593 | ^32^ |
| 58 | butan-1-ol / 1-butyl-3-methylimidazolium bis(trifluoromethylsulfonyl)imide | 26 | Wilhelmy plate | 283.15 - 313.15 | C_10_H_15_F_6_N_3_O_4_S_2_ | 419.36 | 1422.5 - 1451.3 | ^2^ | 0.02352 - 0.03336 | ^27^ |
| 59 | methanol / 1-butyl-3-methylimidazolium bis(trifluoromethylsulfonyl)imide | 24 | Wilhelmy plate | 283.15 - 298.15 | C_10_H_15_F_6_N_3_O_4_S_2_ | 419.36 | 1436.8  - 1451.3 | ^2^ | 0.02641 - 0.03344 | ^27^ |
| 60 | hexan-1-ol / 2-octylisoquinolinium bis((trifluoromethyl)sulfonyl)amide | 24 | Ring tensiometer | 298.15 - 318.15 | C_19_H_24_F_6_N_2_O_4_S_2_ | 522.52 | 1313.6 - 1330.8 | ^33^ | 0.02348 - 0.03187 | ^33^ |
| 61 | oct-1-ene / 1-hexyl-3-methylimidazolium bis[(trifluoromethyl)sulfonyl]imide | 24 | Ring tensiometer | 283.1 -348.15 | C_12_H_19_F_6_N_3_O_4_S_2_ | 447.41 | 1319.5 - 1378.5 | ^34^ | 0.0157 - 0.0312 | ^35^ |
| 62 | water / 3-ethyl-1-methylimidazolium (S)-2-hydroxypropanoate | 22 | digital tensiometer | 298.15 | C_9_H_16_N_2_O_3_ | 200.24 | 1146.1 | ^36^ | 0.05153 - 0.0716 | ^37^ |
| 63 | water / 1-ethyl-3-methylimidazolium octyl sulfate | 21 | Drop volume | 298.15 | C_14_H_28_N_2_O_4_S | 320.45 | 1094.2 | ^38^ | 0.03048 - 0.05692 | ^32^ |
| 64 | methanol / 1-methyl-3-octylimidazolium bis(trifluoromethylsulfonyl)imide | 20 | Wilhelmy plate | 283.15 - 298.15 | C_14_H_23_F_6_N_3_O_4_S_2_ | 475.47 | 1320- 1331 | ^26^ | 0.02505 - 0.03162 | ^27^ |
| 65 | water / 1-ethyl-3-methylimidazolium tetrafluoroborate | 18 | Drop volume | 298.15 | C_6_H_11_BF_4_N_2_ | 197.97 | 1283.3 | ^19^ | 0.05304 - 0.07135 | ^29^ |
| 66 | ethanol / 1-ethyl-3-methylimidazolium octyl sulfate | 18 | Drop volume | 298.15 | C_14_H_28_N_2_O_4_S | 320.45 | 1094.2 | ^38^ | 0.02289 - 0.03091 | ^32^ |
| 67 | water / 3-ethyl-1-methyl-1H-imidazolium hexyl sulfate | 18 | Drop volume | 298.15 | C_12_H_24_N_2_O_4_S | 292.39 | 1130.4 | ^39^ | 0.03477 - 0.06258 | ^32^ |
| 68 | water / 1-ethyl-3-methylimidazolium diethyl phosphate | 16 | Ring tensiometer | 298.15 | C_10_H_21_N_2_O_4_P | 264.26 | 1144.2 | ^40^ | 0.0371 - 0.0719 | ^41^ |
| 69 | water / 1,3-dimethylimidazolium dimethyl phosphate | 16 | Ring tensiometer | 298.15 | C_7_H_15_N_2_O_4_P | 222.18 | 1258.1 | ^42^ | 0.0484 - 0.0719 | ^41^ |
| 70 | water / 1-butyl-3-ethyl-1H-imidazol-3-ium diethyl phosphate | 16 | Ring tensiometer | 298.15 | C_13_H_27_N_2_O_4_P | 306.34 | 1083.21 | ^41^ | 0.0322 - 0.0719 | ^41^ |
| 71 | ethanol / ethylammonium nitrate | 16 | Drop volume | 298.15 | C_2_H_8_N_2_O_3_ | 108.1 | 1212.4 | ^43^ | 0.02362 - 0.04232 | ^44^ |
| 72 | butan-1-ol / 1,3-dimethylimidazolium methylsulfate | 15 | Ring tensiometer | 298.15 | C_6_H_12_N_2_O_4_S | 208.23 | 1329 | ^45^ | 0.02255 - 0.0527 | ^45^ |
| 73 | water / 1-ethyl-3-methylimidazolium ethyl sulfate | 15 | Drop volume | 298.15 | C_8_H_16_N_2_O_4_S | 236.29 | 1237.9 | ^46^ | 0.04716 - 0.07135 | ^32^ |
| 74 | butan-1-ol / 1-ethyl-3-methylimidazolium methyl sulfate | 14 | Ring tensiometer | 293.15 | C_7_H_14_N_2_O_4_S | 222.26 | 1289.48 | ^39^ | 0.02362 - 0.05262 | ^47^ |
| 75 | ethanol / 1-ethyl-3-methylimidazolium methyl sulfate | 14 | Plate tensiometer | 293.15 | C_7_H_14_N_2_O_4_S | 222.26 | 1289.48 | ^39^ | 0.022 - 0.05262 | ^47^ |
| 76 | methanol / 1-ethyl-3-methylimidazolium methyl sulfate | 14 | Plate tensiometer | 293.15 | C_7_H_14_N_2_O_4_S | 222.26 | 1289.48 | ^39^ | 0.0218 - 0.05262 | ^47^ |
| 77 | ethanol / 3-ethyl-1-methyl-1H-imidazolium hexyl sulfate | 13 | Drop volume | 298.15 | C_12_H_24_N_2_O_4_S | 292.39 | 1130.4 | ^39^ | 0.02298 - 0.03477 | ^32^ |
| 78 | butan-1-ol / 1-butyl-3-methylimidazolium bis(trifluoromethylsulfonyl)imide | 13 | Pendant drop shape | 298 | C_10_H_15_F_6_N_3_O_4_S_2_ | 419.36 | 1436.8 | ^2^ | 0.0238 - 0.0328 | ^48^ |
| 79 | ethanol / 1-butyl-3-methylimidazolium tetrafluoroborate | 12 | Drop volume | 298.15 | C_8_H_15_BF_4_N_2_ | 226.03 | 1202.5 | ^2^ | 0.02283 - 0.04533 | ^29^ |
| 80 | methanol / 1-ethyl-3-methylimidazolium dimethylphosphate | 11 | Ring tensiometer | 298.15 | C_8_H_17_N_2_O_4_P | 236.21 | 1217.8 | ^49^ | 0.0224 - 0.0443 | ^41^ |
| 81 | ethanol / 1-butyl-3-methylimidazolium dibutyl phosphate | 11 | Ring tensiometer | 298.15 | C_16_H_33_N_2_O_4_P | 348.42 | 1046.18 | ^41^ | 0.0219 - 0.0294 | ^41^ |
| 82 | ethanol / 1-butyl-3-ethyl-1H-imidazol-3-ium diethyl phosphate | 11 | Ring tensiometer | 298.15 | C_13_H_27_N_2_O_4_P | 306.34 | 1083.21 | ^41^ | 0.0219 - 0.0322 | ^41^ |
| 83 | ethanol / 1,3-diethyl-1H-imidazol-3-ium diethyl phosphate | 11 | Ring tensiometer | 298.15 | C_11_H_23_N_2_O_4_P | 278.29 | 1125.96 | ^41^ | 0.0219 - 0.0355 | ^41^ |
| 84 | ethanol / 1-ethyl-3-methylimidazolium diethyl phosphate | 11 | Ring tensiometer | 298.15 | C_10_H_21_N_2_O_4_P | 264.26 | 1148.86 | ^41^ | 0.0219 - 0.0371 | ^41^ |
| 85 | ethanol / 1-butyl-3-methyl-1H-imidazolium dimethylphosphate | 11 | Ring tensiometer | 298.15 | C_10_H_21_N_2_O_4_P | 264.26 | 1160.1 | ^50^ | 0.0219 - 0.038 | ^41^ |
| 86 | ethanol / 1-ethyl-3-methylimidazolium dimethylphosphate | 11 | Ring tensiometer | 298.15 | C_8_H_17_N_2_O_4_P | 236.21 | 1217.8 | ^49^ | 0.0219 - 0.0443 | ^41^ |
| 87 | ethanol / 1,3-dimethylimidazolium dimethyl phosphate | 11 | Ring tensiometer | 298.15 | C_7_H_15_N_2_O_4_P | 222.18 | 1252.6 | ^41^ | 0.0219 - 0.0484 | ^41^ |
| 88 | methanol / 1-butyl-3-methylimidazolium dibutyl phosphate | 11 | Ring tensiometer | 298.15 | C_16_H_33_N_2_O_4_P | 348.42 | 1046.18 | ^41^ | 0.0224 - 0.0295 | ^41^ |
| 89 | water / 1-ethyl-3-methylimidazolium dimethylphosphate | 11 | Ring tensiometer | 298.15 | C_8_H_17_N_2_O_4_P | 236.21 | 1217.8 | ^49^ | 0.0441 - 0.0719 | ^41^ |
| 90 | water / 1-butyl-3-methyl-1H-imidazolium dimethylphosphate | 11 | Ring tensiometer | 298.15 | C_10_H_21_N_2_O_4_P | 264.26 | 1160.1 | ^50^ | 0.038 - 0.0719 | ^41^ |
| 91 | water / 1,3-diethyl-1H-imidazol-3-ium diethyl phosphate | 11 | Ring tensiometer | 298.15 | C_11_H_23_N_2_O_4_P | 278.29 | 1125.96 | ^41^ | 0.0355 - 0.0719 | ^41^ |
| 92 | water / 1-butyl-3-methylimidazolium dibutyl phosphate | 11 | Ring tensiometer | 298.15 | C_16_H_33_N_2_O_4_P | 348.42 | 1046.18 | ^41^ | 0.0282 - 0.0719 | ^41^ |
| 93 | methanol / 1,3-dimethylimidazolium dimethyl phosphate | 11 | Ring tensiometer | 298.15 | C_7_H_15_N_2_O_4_P | 222.18 | 1252.6 | ^41^ | 0.0224 - 0.0484 | ^41^ |
| 94 | methanol / 1-butyl-3-methyl-1H-imidazolium dimethylphosphate | 11 | Ring tensiometer | 298.15 | C_10_H_21_N_2_O_4_P | 264.26 | 1160.1 | ^50^ | 0.0224 - 0.0381 | ^41^ |
| 95 | methanol / 1-ethyl-3-methylimidazolium diethyl phosphate | 11 | Ring tensiometer | 298.15 | C_10_H_21_N_2_O_4_P | 264.26 | 1148.86 | ^41^ | 0.0224 - 0.0373 | ^41^ |
| 96 | methanol / 1,3-diethyl-1H-imidazol-3-ium diethyl phosphate | 11 | Ring tensiometer | 298.15 | C_11_H_23_N_2_O_4_P | 278.29 | 1125.96 | ^41^ | 0.0224 - 0.0361 | ^41^ |
| 97 | methanol / 1-butyl-3-ethyl-1H-imidazol-3-ium diethyl phosphate | 11 | Ring tensiometer | 298.15 | C_13_H_27_N_2_O_4_P | 306.34 | 1083.21 | ^41^ | 0.0224 - 0.0323 | ^41^ |
| 98 | ethanol / 1-ethyl-3-methylimidazolium ethyl sulfate | 11 | Drop volume | 298.15 | C_8_H_16_N_2_O_4_S | 236.29 | 1237.8 | ^8^ | 0.02201 - 0.04716 | ^32^ |
| 99 | propan-1-ol / 1-butyl-3-methylimidazolium bis(trifluoromethylsulfonyl)imide | 11 | Pendant drop shape | 298 | C_10_H_15_F_6_N_3_O_4_S_2_ | 419.36 | 1436.8 | ^2^ | 0.02334 - 0.0328 | ^48^ |
| 100 | ethanol / 1-methyl-3-octylimidazolium tetrafluoroborate | 10 | Drop volume | 298.15 | C_12_H_23_BF_4_N_2_ | 282.13 | 1104.2 | ^29^ | 0.02333 - 0.03282 | ^29^ |
| 101 | ethanol / 1-hexyl-3-methylimidazolium tetrafluoroborate | 10 | Drop volume | 298.15 | C_10_H_19_BF_4_N_2_ | 254.08 | 1145.4 | ^29^ | 0.02311 - 0.03733 | ^29^ |
| 102 | ethanol / 3-ethyl-1-methylimidazolium butyl sulfate | 10 | Drop volume | 298.15 | C_10_H_20_N_2_O_4_S | 264.34 | 1175.7 | ^32^ | 0.0236 - 0.03962 | ^32^ |
| 103 | water / ethylammonium nitrate | 10 | Drop volume | 298.15 | C_2_H_8_N_2_O_3_ | 108.1 | 1207.8 | ^44^ | 0.04838 - 0.06123 | ^44^ |
| 104 | gamma-butyrolactone / N,N-diethyl-2-methoxy-N-methylethan-1-aminium tetrafluoroborate | 10 | Pt plate | 298.15 | C_8_H_20_BF_4_NO | 233.06 | 1177.4 | ^51^ | 0.0474 - 0.0506 | ^51^ |
| 105 | gamma-butyrolactone / N,N-diethyl-2-methoxy-N-methylethan-1-aminium bis((trifluoromethyl)sulfonyl)amide | 10 | Pt plate | 298.15 | C_10_H_20_F_6_N_2_O_5_S_2_ | 426.39 | 1400.3 | ^51^ | 0.0343 - 0.0382 | ^51^ |
| 106 | ethanol / 1,3-dimethylimidazolium methylsulfate | 9 | Ring tensiometer | 298.15 | C_6_H_12_N_2_O_4_S | 208.23 | 1329 | ^45^ | 0.02155 - 0.0541 | ^45^ |
| 107 | methanol / 1,3-dimethylimidazolium methylsulfate | 9 | Ring tensiometer | 298.15 | C_6_H_12_N_2_O_4_S | 208.23 | 1329 | ^45^ | 0.02209 - 0.0565 | ^45^ |
| 108 | water / n-butylammonium nitrate | 9 | Drop volume | 298.15 | C_4_H_12_N_2_O_3_ | 136.15 | 1105.5 | ^44^ | 0.03317 - 0.04242 | ^44^ |
| 109 | ethanol / n-butylammonium nitrate | 9 | Drop volume | 298.15 | C_4_H_12_N_2_O_3_ | 136.15 | 1105.5 | ^44^ | 0.02407 - 0.03111 | ^44^ |
| 110 | methanol / 1-hexyloxymethyl-3-methylimidazolium tetrafluoroborate | 8 | Ring tensiometer | 308.15 -318.15 | C_11_H_21_BF_4_N_2_O | 284.11 | 1138.7 - 1145.8 | ^52^ | 0.02055 - 0.02113 | ^45^ |
| 111 | butan-1-ol / 1-hexyloxymethyl-3-methylimidazolium tetrafluoroborate | 8 | Ring tensiometer | 308.15 -318.15 | C_11_H_21_BF_4_N_2_O | 284.11 | 1138.7 - 1145.8 | ^52^ | 0.0223 - 0.02319 | ^45^ |
| 112 | hexan-1-ol / 1-hexyloxymethyl-3-methylimidazolium tetrafluoroborate | 8 | Ring tensiometer | 308.15 -318.15 | C_11_H_21_BF_4_N_2_O | 284.11 | 1138.7 - 1145.8 | ^52^ | 0.02302 - 0.02399 | ^45^ |
| 113 | water / 1-hexyl-3-methylimidazolium tetrafluoroborate | 8 | Drop volume | 298.15 | C_10_H_19_BF_4_N_2_ | 254.08 | 1145.4 | ^29^ | 0.03733 - 0.03793 | ^29^ |
| 114 | pyrrole / 1-ethyl-3-methylimidazolium thiocyanate | 7 | Plate tensiometer | 298.15 | C_7_H_11_N_3_S | 169.25 | 1117 | ^53^ | 0.0409 - 0.0474 | ^53^ |
| 115 | pyridine / 1-ethyl-3-methylimidazolium thiocyanate | 7 | Plate tensiometer | 298.15 | C_7_H_11_N_3_S | 169.25 | 1117 | ^53^ | 0.0374 - 0.0465 | ^53^ |
| 116 | quinoline / 1-ethyl-3-methylimidazolium thiocyanate | 7 | Plate tensiometer | 298.15 | C_7_H_11_N_3_S | 169.25 | 1117 | ^53^ | 0.0295 - 0.0408 | ^53^ |
| 117 | indoline / 1-ethyl-3-methylimidazolium thiocyanate | 7 | Plate tensiometer | 298.15 | C_7_H_11_N_3_S | 169.25 | 1117 | ^53^ | 0.0399 - 0.044 | ^53^ |
| 118 | thiophene / 1-ethyl-3-methylimidazolium thiocyanate | 7 | Plate tensiometer | 298.15 | C_7_H_11_N_3_S | 169.25 | 1117 | ^53^ | 0.0308 - 0.0455 | ^53^ |
| 119 | methanol / 1-butyl-3-methylimidazolium octyl sulfate | 7 | Ring tensiometer | 298.15 | C_16_H_32_N_2_O_4_S | 348.5 | 1068 | ^45^ | 0.02444 - 0.02837 | ^45^ |
| 120 | butan-1-ol / 1-butyl-3-methylimidazolium octyl sulfate | 7 | Ring tensiometer | 298.15 | C_16_H_32_N_2_O_4_S | 348.5 | 1068 | ^45^ | 0.0236 - 0.02513 | ^45^ |
| 121 | ethanol / 1-butyl-3-methylimidazolium methyl sulfate | 7 | Ring tensiometer | 298.15 | C_9_H_18_N_2_O_4_S | 250.31 | 1212 | ^45^ | 0.0225 - 0.0414 | ^45^ |
| 122 | butan-1-ol / 1-butyl-3-methylimidazolium methyl sulfate | 6 | Ring tensiometer | 298.15 | C_9_H_18_N_2_O_4_S | 250.31 | 1212 | ^45^ | 0.02265 - 0.02836 | ^45^ |
| 123 | methanol / 1-butyl-3-methylimidazolium methyl sulfate | 6 | Ring tensiometer | 298.15 | C_9_H_18_N_2_O_4_S | 250.31 | 1212 | ^45^ | 0.02329 - 0.03803 | ^45^ |
| 124 | butan-1-ol / 1-octylpyridinium nitrate | 6 | digital tensiometer | 298.15 | C_13_H_22_N_2_O_3_ | 254.33 | 1089.5 | ^54^ | 0.02367 - 0.02753 | ^54^ |
| 125 | ethanol / 1-octylpyridinium nitrate | 5 | digital tensiometer | 298.15 | C_13_H_22_N_2_O_3_ | 254.33 | 1089.5 | ^54^ | 0.02155 - 0.02975 | ^54^ |
| 126 | methanol / 1-octylpyridinium nitrate | 5 | digital tensiometer | 298.15 | C_13_H_22_N_2_O_3_ | 254.33 | 1089.5 | ^54^ | 0.02209 - 0.032 | ^54^ |
| 127 | ethanol / 1-ethyl-3-methylimidazolium tetrafluoroborate | 5 | Drop volume | 298.15 | C_6_H_11_BF_4_N_2_ | 197.97 | 1283.3 | ^19^ | 0.03358 - 0.05304 | ^29^ |

**References**

1 Dong, Q. *et al.* ILThermo: A free-access web database for thermodynamic properties of ionic liquids. *Journal of Chemical & Engineering Data* **52**, 1151-1159 (2007).

2 Salgado, J. *et al.* Density and viscosity of three (2, 2, 2-trifluoroethanol+ 1-butyl-3-methylimidazolium) ionic liquid binary systems. *The Journal of Chemical Thermodynamics* **70**, 101-110 (2014).

3 Li, Z. *et al.* Surface tension of binary mixtures of (ionic liquid+ tributyl phosphate). *The Journal of Chemical Thermodynamics* **132**, 214-221 (2019).

4 Freire, M. G. *et al.* Thermophysical characterization of ionic liquids able to dissolve biomass. *Journal of Chemical & Engineering Data* **56**, 4813-4822 (2011).

5 Castro, M. C., Rodríguez, H., Arce, A. & Soto, A. Mixtures of ethanol and the ionic liquid 1-ethyl-3-methylimidazolium acetate for the fractionated solubility of biopolymers of lignocellulosic biomass. *Industrial & Engineering Chemistry Research* **53**, 11850-11861 (2014).

6 Castro, M. C., Arce, A., Soto, A. & Rodríguez, H. Thermophysical characterization of the mixtures of the ionic liquid 1-ethyl-3-methylimidazolium acetate with 1-propanol or 2-propanol. *Journal of Chemical & Engineering Data* **61**, 2299-2310 (2016).

7 Fang, D.-W., Hu, X.-H., Liang, K.-H., Yan, Q. & Wei, J. The excess molar volume and the molar surface Gibbs energy of the binary of the ether-functionalized ionic liquids [C22O1IM][TfO] with ethanol and isomeric propanols at T=(288.15–318.15) K. *Thermochimica Acta* **682**, 178383 (2019).

8 Gacino, F. M., Regueira, T., Lugo, L., Comunas, M. J. & Fernandez, J. Influence of molecular structure on densities and viscosities of several ionic liquids. *Journal of Chemical & Engineering Data* **56**, 4984-4999 (2011).

9 Fang, D. *et al.* Insight into the solute-solvent interactions by physicochemical and excess properties in binary systems of the ether-and allyl-based functionalized ionic liquids with acetonitrile. *Journal of the Taiwan Institute of Chemical Engineers* **133**, 104275 (2022).

10 Nonthanasin, T., Henni, A. & Saiwan, C. Densities and low pressure solubilities of carbon dioxide in five promising ionic liquids. *RSC Advances* **4**, 7566-7578 (2014).

11 Wei, J. *et al.* Effect of temperature on the minimum excess molar volume and the molar surface Gibbs energy of the binary of the ether-functionalized ionic liquids [C22O1IM][SCN] with monohydric alcohols at T=(288.15–318.15) K. *Journal of Molecular Liquids* **307**, 112856 (2020).

12 Zhang, Q., Li, Q., Liu, D., Zhang, X. & Lang, X. Density, dynamic viscosity, electrical conductivity, electrochemical potential window, and excess properties of ionic liquid N-butyl-pyridinium dicyanamide and binary system with propylene carbonate. *Journal of Molecular Liquids* **249**, 1097-1106 (2018).

13 Xing, N., Liang, K., Fang, D., Guan, W. & Yang, J. The molar surface Gibbs energy and its application to the binary mixtures of N-butylpyridinium dicyanamide [C4py][DCA] with alcohols. *The Journal of Chemical Thermodynamics* **128**, 283-294 (2019).

14 Bandrés, I., Royo, F. M., Gascón, I., Castro, M. & Lafuente, C. Anion influence on thermophysical properties of ionic liquids: 1-butylpyridinium tetrafluoroborate and 1-butylpyridinium triflate. *The Journal of Physical Chemistry B* **114**, 3601-3607 (2010).

15 Antón, V., Artigas, H., Muñoz-Embid, J., Artal, M. & Lafuente, C. Thermophysical properties of two binary aqueous mixtures containing a pyridinium-based ionic liquid. *The Journal of Chemical Thermodynamics* **99**, 116-123 (2016).

16 Bandres, I., López, M. C., Castro, M., Barbera, J. & Lafuente, C. Thermophysical properties of 1-propylpyridinium tetrafluoroborate. *The Journal of Chemical Thermodynamics* **44**, 148-153 (2012).

17 García-Mardones, M., Cea, P., Gascón, I. & Lafuente, C. Thermodynamic study of the surface of liquid mixtures containing pyridinium-based ionic liquids and alkanols. *The Journal of Chemical Thermodynamics* **78**, 234-240 (2014).

18 Bandrés, I., Giner, B., Artigas, H., Royo, F. M. & Lafuente, C. Thermophysic comparative study of two isomeric pyridinium-based ionic liquids. *The Journal of Physical Chemistry B* **112**, 3077-3084 (2008).

19 Neves, C. M. *et al.* Systematic study of the thermophysical properties of imidazolium-based ionic liquids with cyano-functionalized anions. *The Journal of Physical Chemistry B* **117**, 10271-10283 (2013).

20 Shojaeian, A. Surface tension measurements of aqueous 1-alkyle-3-methylimidazolume tetrafluoroborate {[Cnmim][BF4](n= 2, 4, 6)} solutions and modeling surface tension of ionic liquid binary mixtures using six various models. *Thermochimica Acta* **673**, 119-128 (2019).

21 Geppert-Rybczyńska, M., Lehmann, J. K., Safarov, J. & Heintz, A. Thermodynamic surface properties of [BMIm][NTf2] or [EMIm][NTf2] binary mixtures with tetrahydrofuran, acetonitrile or dimethylsulfoxide. *The Journal of Chemical Thermodynamics* **62**, 104-110 (2013).

22 Jiang, H. *et al.* Density and surface tension of pure ionic liquid 1-butyl-3-methyl-imidazolium l-lactate and its binary mixture with alcohol and water. *The Journal of Chemical Thermodynamics* **64**, 1-13 (2013).

23 Domańska, U. & Królikowska, M. Effect of temperature and composition on the surface tension and thermodynamic properties of binary mixtures of 1-butyl-3-methylimidazolium thiocyanate with alcohols. *Journal of colloid and interface science* **348**, 661-667 (2010).

24 Fröba, A. P., Kremer, H. & Leipertz, A. Density, refractive index, interfacial tension, and viscosity of ionic liquids [EMIM][EtSO4],[EMIM][NTf2],[EMIM][N (CN) 2], and [OMA][NTf2] in dependence on temperature at atmospheric pressure. *The Journal of Physical Chemistry B* **112**, 12420-12430 (2008).

25 Vakili-Nezhaad, G., Vatani, M., Asghari, M. & Ashour, I. Effect of temperature on the physical properties of 1-butyl-3-methylimidazolium based ionic liquids with thiocyanate and tetrafluoroborate anions, and 1-hexyl-3-methylimidazolium with tetrafluoroborate and hexafluorophosphate anions. *The Journal of Chemical Thermodynamics* **54**, 148-154 (2012).

26 Papović, S., Bešter-Rogač, M., Vraneš, M. & Gadžurić, S. The effect of the alkyl chain length on physicochemical features of (ionic liquids+ γ-butyrolactone) binary mixtures. *The Journal of Chemical Thermodynamics* **99**, 1-10 (2016).

27 Andreatta, A. E., Rodil, E., Arce, A. & Soto, A. Surface tension of binary mixtures of 1-alkyl-3-methyl-imidazolium bis (trifluoromethylsulfonyl) imide ionic liquids with alcohols. *Journal of Solution Chemistry* **43**, 404-420 (2014).

28 Geppert-Rybczyńska, M., Lehmann, J. K. & Heintz, A. Physicochemical properties of two 1-alkyl-1-methylpyrrolidinium bis [(trifluoromethyl) sulfonyl] imide ionic liquids and of binary mixtures of 1-butyl-1-methylpyrrolidinium bis [(trifluoromethyl) sulfonyl] imide with methanol or acetonitrile. *The Journal of Chemical Thermodynamics* **71**, 171-181 (2014).

29 Rilo, E., Pico, J., García-Garabal, S., Varela, L. & Cabeza, O. Density and surface tension in binary mixtures of CnMIM-BF4 ionic liquids with water and ethanol. *Fluid Phase Equilibria* **285**, 83-89 (2009).

30 Domańska, U., Lukoshko, E. V. & Królikowski, M. Separation of thiophene from heptane with ionic liquids. *The Journal of Chemical Thermodynamics* **61**, 126-131 (2013).

31 Domańska, U., Królikowska, M. & Walczak, K. Density, viscosity and surface tension of binary mixtures of 1-butyl-1-methylpyrrolidinium tricyanomethanide with benzothiophene. *Journal of Solution Chemistry* **43**, 1929-1946 (2014).

32 Rilo, E., Domínguez-Pérez, M., Vila, J., Varela, L. & Cabeza, O. Surface tension of four binary systems containing (1-ethyl-3-methyl imidazolium alkyl sulphate ionic liquid+ water or+ ethanol). *The Journal of Chemical Thermodynamics* **49**, 165-171 (2012).

33 Domańska, U., Zawadzki, M. & Lewandrowska, A. Effect of temperature and composition on the density, viscosity, surface tension, and thermodynamic properties of binary mixtures of N-octylisoquinolinium bis {(trifluoromethyl) sulfonyl} imide with alcohols. *The Journal of Chemical Thermodynamics* **48**, 101-111 (2012).

34 Fröba, A. *et al.* Thermal conductivity of ionic liquids: Measurement and prediction. *International Journal of Thermophysics* **31**, 2059-2077 (2010).

35 Ahosseini, A., Sensenich, B., Weatherley, L. R. & Scurto, A. M. Phase equilibrium, volumetric, and interfacial properties of the ionic liquid, 1-hexyl-3-methylimidazolium bis (trifluoromethylsulfonyl) amide and 1-octene. *Journal of Chemical & Engineering Data* **55**, 1611-1617 (2010).

36 Machida, H., Taguchi, R., Sato, Y. & Smith Jr, R. L. Measurement and correlation of high pressure densities of ionic liquids, 1-Ethyl-3-methylimidazolium l-Lactate ([emim][Lactate]), 2-Hydroxyethyl-trimethylammonium l-Lactate ([(C2H4OH)(CH3) 3N][Lactate]), and 1-Butyl-3-methylimidazolium chloride ([bmim][Cl]). *Journal of Chemical & Engineering Data* **56**, 923-928 (2010).

37 Wang, J.-y., Jiang, H.-c., Liu, Y.-m. & Hu, Y.-q. Density and surface tension of pure 1-ethyl-3-methylimidazolium L-lactate ionic liquid and its binary mixtures with water. *The Journal of Chemical Thermodynamics* **43**, 800-804 (2011).

38 Hasse, B. *et al.* Viscosity, interfacial tension, density, and refractive index of ionic liquids [EMIM][MeSO3],[EMIM][MeOHPO2],[EMIM][OcSO4], and [BBIM][NTf2] in dependence on temperature at atmospheric pressure. *Journal of Chemical & Engineering Data* **54**, 2576-2583 (2009).

39 Costa, A. J., Esperanca, J. M., Marrucho, I. M. & Rebelo, L. P. N. Densities and viscosities of 1-ethyl-3-methylimidazolium n-alkyl sulfates. *Journal of Chemical & Engineering Data* **56**, 3433-3441 (2011).

40 Zorębski, E., Musiał, M., Bałuszyńska, K., Zorębski, M. & Dzida, M. Isobaric and isochoric heat capacities as well as isentropic and isothermal compressibilities of di-and trisubstituted imidazolium-based ionic liquids as a function of temperature. *Industrial & Engineering Chemistry Research* **57**, 5161-5172 (2018).

41 Ren, N.-n., Gong, Y.-h., Lu, Y.-z., Meng, H. & Li, C.-x. Surface tension measurements for seven imidazolium-based dialkylphosphate ionic liquids and their binary mixtures with water (methanol or ethanol) at 298.15 K and 1 atm. *Journal of Chemical & Engineering Data* **59**, 189-196 (2014).

42 Sanchez, P. B., González, B., Salgado, J., Padua, A. A. & Garcia, J. Cosolvent effect on physical properties of 1, 3-dimethyl imidazolium dimethyl phosphate and some theoretical insights on cellulose dissolution. *Journal of Molecular Liquids* **265**, 114-120 (2018).

43 Canongia Lopes, J. N. *et al.* Protonic ammonium nitrate ionic liquids and their mixtures: Insights into their thermophysical behavior. *The Journal of Physical Chemistry B* **120**, 2397-2406 (2016).

44 Segade, L. *et al.* Surface and bulk characterisation of mixtures containing alkylammonium nitrates and water or ethanol: Experimental and simulated properties at 298.15 K. *Journal of Molecular Liquids* **222**, 663-670 (2016).

45 Domańska, U., Pobudkowska, A. & Rogalski, M. Surface tension of binary mixtures of imidazolium and ammonium based ionic liquids with alcohols, or water: Cation, anion effect. *Journal of colloid and interface science* **322**, 342-350 (2008).

46 de Castro, C. A. N. *et al.* Studies on the density, heat capacity, surface tension and infinite dilution diffusion with the ionic liquids [C4mim][NTf2],[C4mim][dca],[C2mim][EtOSO3] and [Aliquat][dca]. *Fluid Phase Equilibria* **294**, 157-179 (2010).

47 Wang, J.-Y., Zhao, F.-Y., Liu, Y.-M., Wang, X.-L. & Hu, Y.-Q. Thermophysical properties of pure 1-ethyl-3-methylimidazolium methylsulphate and its binary mixtures with alcohols. *Fluid Phase Equilibria* **305**, 114-120 (2011).

48 Wandschneider, A., Lehmann, J. K. & Heintz, A. Surface tension and density of pure ionic liquids and some binary mixtures with 1-propanol and 1-butanol. *Journal of Chemical & Engineering Data* **53**, 596-599 (2008).

49 Vataščin, E. & Dohnal, V. Phase equilibria and energetics of binary mixtures of water with highly hydrophilic [EMIM]-based ionic liquids: Methanesulfonate, methylsulfate, and dimethylphosphate. *Fluid Phase Equilibria* **521**, 112659 (2020).

50 Martins, M. A. *et al.* Selection and characterization of non-ideal ionic liquids mixtures to be used in CO2 capture. *Fluid Phase Equilibria* **518**, 112621 (2020).

51 Zhang, Q., Feng, S., Zhang, X. & Wei, Y. Thermodynamic properties and intermolecular interactions of ionic liquids [DEME][BF4] or [DEME][TFSI] and their binary mixture systems with GBL. *Journal of Molecular Liquids* **328**, 115373 (2021).

52 Domańska, U., Pobudkowska, A. & Bocheńska, P. Extraction of nitrofurantoin using ionic liquids. *Journal of Chemical & Engineering Data* **57**, 1894-1898 (2012).

53 Anantharaj, R. & Banerjee, T. Phase behaviour of 1-ethyl-3-methylimidazolium thiocyanate ionic liquid with catalytic deactivated compounds and water at several temperatures: experiments and theoretical predictions. *International Journal of Chemical Engineering* **2011** (2011).

54 Jiang, H., Wang, J., Zhao, F., Qi, G. & Hu, Y. Volumetric and surface properties of pure ionic liquid n-octyl-pyridinium nitrate and its binary mixture with alcohol. *The Journal of Chemical Thermodynamics* **47**, 203-208 (2012).
